# Supplementary material for: Mycorrhizal symbioses and tree diversity in global forest communities
Source: Sci Adv. 2025 Jun 13;11(24):eadt5743. doi: 10.1126/sciadv.adt5743 (PMC12164979; doi:10.1126/sciadv.adt5743)
Supplement: 20250613-1 [file sciadv.adt5743.v1.pdf]

ECOLOGY

Mycorrhizal symbioses and tree diversity in global forest communities

Copyright © 2025 The Authors, some rights reserved; exclusive licensee American Association for the Advancement of Science. No claim to original U.S. Government Works. Distributed under a Creative Commons Attribution License 4.0 (CC BY).

Feng Jiang[1]†, Xucai Pu[1]†, Bernhard Schmid[1,2], Peter B. Reich[3,4,5], Jingjing Liang[6]*, Akane O. Abbasi[6], Jesús Aguirre-Gutiérrez[7,8], Angelica Maria Almeyda Zambrano[9], Jan Altman[10,11], Juan Gabriel Álvarez-González[12], Luciana F. Alves[13], Bienvenu H. K. Amani[14,15], Christian Ammer[16], Gerardo A. Aymard[17,18], Naveen Babu Kanda[19], Meredith L. Bastian[20,21], Jean-Francois Bastin[22], Marijn Bauters[23], Pascal Boeckx[24], Svetlana N. Bondarchuk[25], Alexander Bondarev[26], Francis Q. Brearley[27], Sophie Brennan[28], Jaime Briseño-Reyes[29], Eben N. Broadbent[30], Goran Češljar[31], Han Y. H. Chen[32,33], Chelsea Chisholm[34], WookJin Choi[6], Emil Cienciala[35,36], Connie J. Clark[37], Alessio Collalti[38,39], José Javier Corral-Rivas[29], Javid Ahmad Dar[40,41], Selvadurai Dayanandan[42], Sergio de-Miguel[43,44], Ashaq Ahmad Dar[45], Géraldine Derroire[46], Ilija Djordjevic[47], Tran Van Do[48], Jiří Doležal[10,49], Aurélie Dourdain[46], Teresa Eyre[50], Adandé Belarmain Fandohan[51], Lorenzo Frizzera[52], Roberto Cazzolla Gatti[53], Damiano Gianelle[52], M. Socorro González Elizondo[54], Elisa Grieco[38], David J. Harris[55], Andy Hector[56], Bruno Hérault[57,58], Cang Hui[59,60], Nobuo Imai[61], Andrzej M. Jagodziński[62,63], Chengjun Ji[1], Lin Jiang[64], Carlos A. Joly[65,66], Viktor N. Karminov[67], Kuswata Kartawinata[68]‡, Justin N. Kassi[69], Elizabeth Kearsley[23], Gunnar Keppel[70], Mohammed Latif Khan[71], Carine Klauberg[72], Kirill A. Korznikov[10,73], Subashree Kothandaraman[40,41], Florian Kraxner[74], Leonid Krivobokov[75], Dmitry Kucher[76], Amit Kumar[77,78], Anna Kvashnina[79], Gaia Vaglio Laurin[80], Rodrigo Vieira Leite[81,82], Moses B. Libalah[83,84], Ekaterina S. Lonkina[85], Huicui Lu[86], Shan Luo[87,88], Yuan Luo[1], Emma Mackintosh[89], Andrew R. Marshall[90,91], Rodolfo Vásquez Martínez[92], Radim Matula[93], William McDonald[94], Ayyappan Narayanan[19], María Guadalupe Nava-Miranda[95,96], Jagadeesan Naveenkumar[45], Abel Monteagudo Mendoza[92,97], Stanisław Miścicki[98], Tatyana Moskalyuk[99], Liudmila Mukhortova[75], Sharif A. Mukul[100,101], Gert-Jan Nabuurs[102,103], Victor J. Neldner[50], Radovan Nevenic[104], Anny E. N'Guessan[105], Michael Ngugi[50], Alain Paquette[106], Elena I. Parfenova[75], Marc Parren[103], Narayanaswamy Parthasarathy[45], Pablo L. Peri[107], Sebastian Pfautsch[108], Maria T. F. Piedade[109], Galina Polyakova[75], Axel Dalberg Poulsen[55], John R. Poulsen[110], Hans Pretzsch[111], Mirco Rodeghiero[52], Ervan Rutishauser[112], Purabi Saikia[113], Philippe Saner[114], Dmitry Schepaschenko[74], Jochen Schöngart[115], Eric B. Searle[32], Douglas Sheil[103], Zehao Shen[1], Stephanie Shooner[116,117], Anatoly Shvidenko[74,75], Carlos A. Silva[118], Plinio Sist[119], Ferry Slik[120], Wenqi Song[1], Alexandre F. Souza[121], Krzysztof Stereńczak[122], Somaiah Sundarapandian[45], Martin Svátek[123], Miroslav Svoboda[93], Zhiyao Tang[1], Natalia Targhetta[115], Nadja Tchebakova[75], Elena Tikhonova[124], Liam Trethowan[125], Daniel José Vega-Nieva[29], Hans Verbeeck[23], Simone A. Vieira[126], Camille Volle[127], Anna S. Vozmishcheva[128], Foma K. Vozmitel[79,129], Hua-Feng Wang[130], Shaopeng Wang[1], Xiangping Wang[131], Florian Wittmann[132], Chengyang Zheng[1], Biao Zhu[1], Irié Casimir Zo-Bi[133], Jingyun Fang[1,134], Zhiheng Wang[1]*

Unraveling the mechanisms underlying the maintenance of species diversity is a central pursuit in ecology. It has been hypothesized that ectomycorrhizal (EcM) in contrast to arbuscular mycorrhizal fungi can reduce tree species diversity in local communities, which remains to be tested at the global scale. To address this gap, we analyzed global forest inventory data and revealed that the relationship between tree species richness and EcM tree proportion varied along environmental gradients. Specifically, the relationship is more negative at low latitudes and in moist conditions but is unimodal at high latitudes and in arid conditions. The negative association of EcM tree proportion on species diversity at low latitudes and in humid conditions is likely due to more negative plant-soil microbial interactions in these regions. These findings extend our knowledge on the mechanisms shaping global patterns in plant species diversity from a belowground view.

INTRODUCTION

Forests contain the highest biomass among the terrestrial ecosystems and are critical for both the mitigation of climate change and biodiversity conservation (*1*). Yet, severe anthropogenic disturbances have led to high risks of die-offs and even extinctions of tree species and substantial loss of forests at the global scale (*2*–*4*). Understanding the mechanisms underpinning tree species diversity in forest ecosystems is fundamental for the conservation of tree species diversity, and, hence, has intrigued ecologists in the past few decades. In general, species diversity in local forest communities has been associated with factors such as temperature (*5*), rainfall (*6*, *7*), and soil fertility (*8*), as well as negative density dependence (*9*, *10*). In contrast, the role of mutualistic interactions in mediating tree species diversity has rarely been evaluated (*11*), although mutualistic biological interaction networks are also facing high risks of decline due to global changes (*12*). Understanding the role of mutualistic biological interactions in maintaining tree diversity can facilitate the protection and restoration of forest ecosystems. Mycorrhizal symbioses are probably the most widely occurring mutualistic interactions in terrestrial ecosystems, in which fungal partners provide plants with soil nutrients and protection against soil-borne pathogens in return for carbon (*13*, *14*). It is conceivable that such mutualistic interactions by mycorrhizal symbioses can alleviate the effects of negative biotic interactions (*15*), in particular, negative plant-soil feedbacks. Therefore, mycorrhizal fungi are expected to weaken negative density dependence of tree species and thus reduce tree species diversity in forest communities (*16*, *17*). Yet, the role of mycorrhizal fungi in the maintenance of tree species diversity remains to be evaluated.

Recent studies suggest that the alleviation of negative plant-soil feedbacks varies between two main types of mycorrhizal fungi associated with tree species: ectomycorrhizal (EcM) and arbuscular mycorrhizal (AM) fungi (*18*). Experiments and field observations have found that EcM association can more strongly alleviate negative plant-soil feedbacks (*17*, *19*) and hence conspecific negative density dependence in forest communities (*16*, *20*–*24*) than AM association. These distinctions are presumed to stem from the greater host specificity of EcM fungi (*25*) and their capacity to provide superior physical protection against soil-borne pathogens by forming a mantle around root tips of trees (*11*, *17*). Consequently, a higher proportion of EcM trees in forest communities is expected to lead to a lower tree species richness (*11*, *26*, *27*) (termed as the EcM dominance hypothesis hereafter, Fig. 1A). In contrast, the theory of niche partitioning predicts that tree species richness will be higher in communities with a mixture of both mycorrhizal types than in those dominated by either AM or EcM trees (*28*–*30*). Niche partitioning between EcM and AM tree species is expected because of their abilities to access different pools of soil nutrients (*31*, *32*). Specifically, EcM fungi can directly access soil organic nutrients by exuding extracellular enzymes (*33*), whereas AM fungi have a greater affinity to take up soil inorganic nutrients due to their limited enzymatic capabilities (*34*). In addition, functional traits of plant leaves and roots (e.g., nitrogen content) are usually different between EcM and AM tree species (*35*, *36*). These differences in plant functional traits can increase the partitioning of nutrient uptake strategies between EcM and AM trees, thereby promoting plant species coexistence and leading to higher species richness in communities with mixed mycorrhizal types (*19*, *26*) (termed as the mycorrhizal mixture hypothesis hereafter, Fig. 1B).

Although the EcM dominance hypothesis has been proposed repeatedly since the last century (*11*, *26*, *27*, *37*), empirical studies directly evaluating the relationship between tree species richness and the relative proportion of EcM and AM trees are rare (*21*, *29*). Specifically, a recent study conducted in the USA revealed a unimodal relationship between tree species richness and EcM tree proportion, challenging the EcM dominance hypothesis while supporting the mycorrhizal mixture hypothesis (*29*). This finding raises questions about the mechanisms by which the mutualistic interactions between plants and mycorrhizal fungi drive tree species coexistence and species diversity within forest communities, as revealed by recent plant-soil feedback experiments (*17*, *19*) and seedling dynamics analyses in forests (*16*, *21*–*24*). To reconcile the inconsistencies between the longstanding EcM dominance hypothesis and the recently supported mycorrhizal mixture hypothesis, we propose that these two hypotheses may not be mutually exclusive, and the alleviation of negative plant-soil feedbacks by EcM fungi and niche partitioning between EcM and AM tree species may have jointly determined tree species richness. If so, we expect an asymmetrical unimodal relationship between the proportion of EcM trees and tree species richness. Consequently, tree species richness would peak in communities featuring a mixture of both mycorrhizal types but would be comparatively lower in EcM-dominated than AM-dominated forests (termed as the integrated hypothesis hereafter, Fig. 1C) (*29*).

Because biotic interactions usually vary along environmental gradients (*38*–*40*), we anticipate that the support for each hypothesis (Fig. 1, A to C) may vary depending on environmental conditions. At low latitudes and in moist conditions, where biotic stresses such as pathogens and herbivory are likely stronger (*40*, *41*), the alleviation of negative plant-soil feedbacks by EcM fungi would be more pronounced. Thus, the EcM dominance hypothesis may be better supported in these conditions. In contrast, at high latitudes or in arid conditions, niche partitioning may become more important in shaping tree species richness because environmental stress may lead to decreased competition and increased cooperation (*42*). Last, at mid-latitudes and in moderately moist conditions, the integrated hypothesis may be more supported.

If mycorrhizal associations interact with environmental factors to influence plant diversity as we hypothesized, regional-scale analyses may fail to detect these interactions or may even reveal opposing trends of plant diversity along the gradient of the proportion of EcM trees (*21*, *29*). Therefore, a global-scale evaluation is needed to reconcile recent regional findings and predictions of the longstanding EcM dominance hypothesis (*21*, *29*). To test the relative roles of these hypotheses in different forests (Fig. 1, A to C), we used a dataset of global forest inventory plots (Fig. 1D) to evaluate the relationships between the proportion of EcM trees and species richness in local forest communities across gradients of latitudes and aridity. Because tree species richness and the proportion of EcM trees may respond similarly to environmental gradients on a global scale, our analyses also controlled for these potential environmental factors before testing the relationships between tree species richness and the proportion of EcM trees.

RESULTS

To assess the relationship between the proportion of EcM trees and species richness in forest communities, we used a generalized

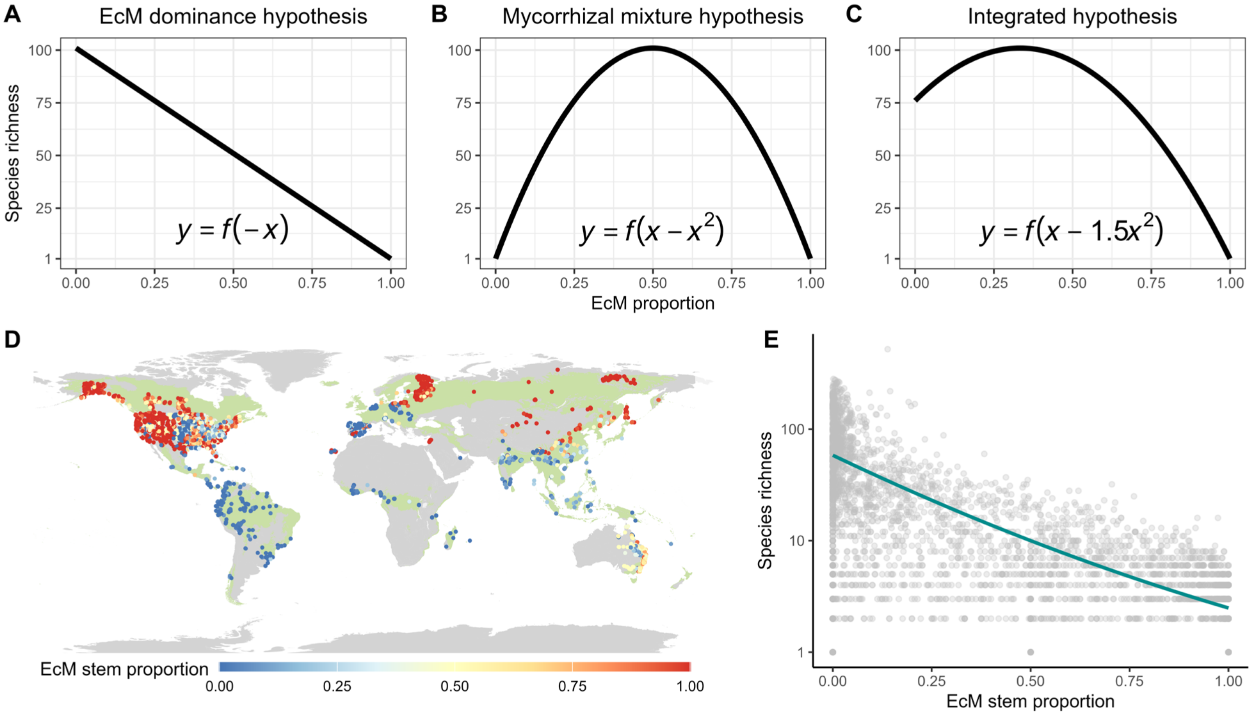

Fig. 1. Hypothetical relationships between the proportion of EcM tree individuals in a community and tree species richness; and forest plot data used to test the predictions. (**A** to **C**) Species richness can show a negative (only includes a negative first order term, hypothesis 1, or EcM dominance hypothesis), symmetrically unimodal (includes both linear and quadratic terms but the absolute coefficients of both terms have similar magnitude, hypothesis 2, or mycorrhizal mixture hypothesis), or asymmetrically unimodal (the absolute value of quadratic term being larger than that of the first-order term, hypothesis 3, or integrated hypothesis) relationship with the proportion of EcM trees. (**D**) The global pattern of the proportion of EcM tree species based on stem abundance in our forest plot dataset (N = 4090). The green background on the map indicates forest regions classified by (*43*). (**E**) Raw relationship between the proportion of EcM tree individuals and tree species richness using the generalized linear model without controlling for covariates (r^2 = 0.59).

linear model with a log link and a negative binomial distribution of model residuals. To reduce the potential impacts of different sampling efforts across regions on our analysis, we down-sampled the global forest plots (N = 442,384; fig. S1) to 4090 plots (see Materials and Methods; Fig. 1D). In the down-sampled dataset, 579 plots were distributed in boreal forests, 2457 in temperate forests, and 1054 in tropical forests. Communities with a low proportion of EcM trees (high proportion of AM trees; fig. S3A) had a wide range of species richness ranging from very low to very high, whereas communities with a high proportion of EcM trees consistently had relatively low tree species richness (Fig. 1E and fig. S3B). Because tree species richness was also related to climatic, topographical, soil, and inventory-survey variables (Fig. 2A), we also included these potential covariates into our statistical models. We found significant interactions between the proportion of EcM trees and absolute latitude and the aridity index in their association with tree species richness (P < 0.001; Fig. 2, A to D, and table S2). Moving from low to high latitudes or from moist to arid conditions (especially at middle to high latitudes), the relationships between tree species richness and EcM tree proportion changed gradually from negative to unimodal (Fig. 2, B to D, and fig. S4). Our results did not change systematically when EcM tree proportion was quantified by basal area instead of the number of tree individuals, and species diversity within communities was quantified by Shannon and Simpson indices instead of tree species richness (see fig. S5).

To further account for other potential variables influencing tree species richness across latitudinal regions, such as geographic history and variation in species pool size across regions, we conducted similar generalized linear model analyses within each biome and ecoregion using all forest plots (N = 442,384) (*43*). An ecoregion is a geographic unit within a biome with limited extent and characterized by similar climate, soil, vegetation, and geographic history (*43*). The latitudinal variation in the relationships between EcM tree proportion and tree species richness across different biomes and ecoregions was similar to that shown in the global analysis. Specifically, the relationships changed from negative in biomes or ecoregions at low latitudes to unimodal or positively unimodal in biomes or ecoregions at high latitudes (figs. S6 and S7). For instance, in tropical and subtropical moist broadleaved forests, tree species richness exhibited a negative correlation with EcM tree proportion (linear EcM = −1.58 and quadratic EcM = −0.38). Conversely, in boreal forests, tree species richness displayed a positively unimodal relationship with EcM tree proportion (linear EcM = 13.05 and quadratic EcM = −8.63). Although not statistically significant, the relationships between EcM tree proportion and tree species richness across different biomes and ecoregions tended to be negatively unimodal in moist conditions and positively unimodal in arid conditions (figs. S6 and S7).

To evaluate the relative importance of EcM tree proportion on species richness compared with other covariates, we used random forest models at the global and regional scales. At the global scale,

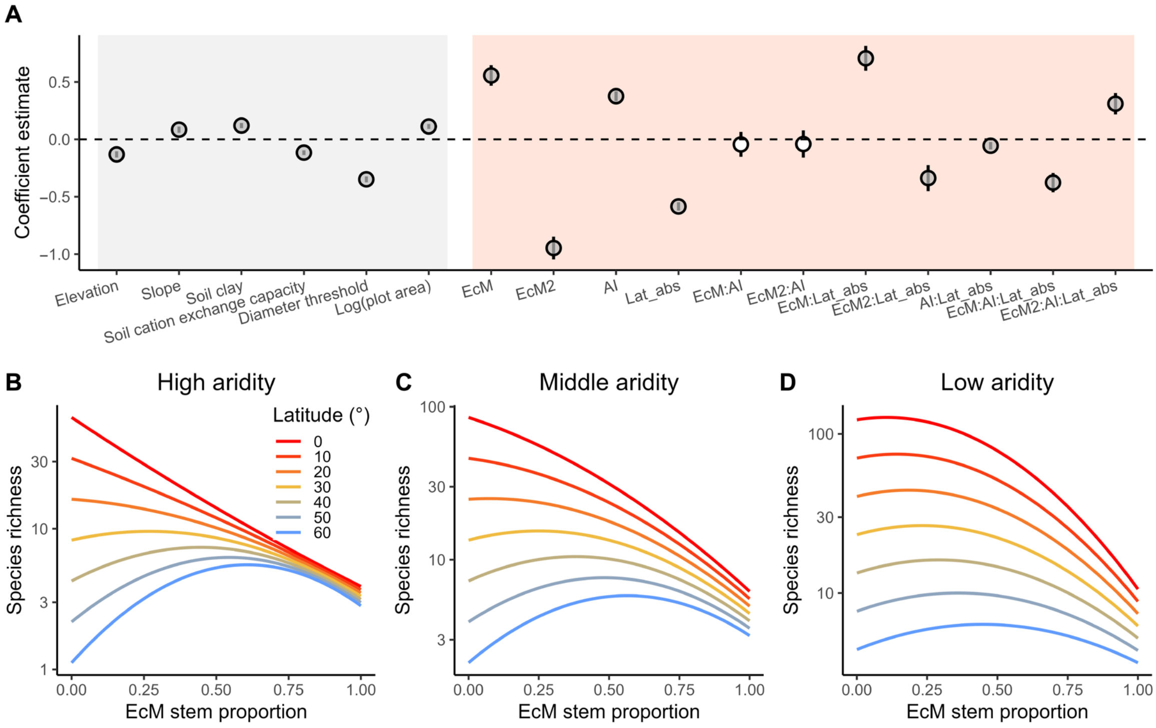

Fig. 2. Effects of EcM tree proportion interacting with absolute latitude and aridity on tree species richness. (**A**) Standardized coefficients of generalized linear model, open and solid circles indicate nonsignificant and significant relationships ($P < 0.05$); (**B** to **D**) relationships between EcM tree proportion and tree species richness across gradients of absolute latitudes and aridity (aridity index = 0.5, 1.2, and 2); lines are predicted species richness ($\log_{10}$ back-transformed) after controlling for covariates; the ranges of species richness differ among these panels due to variations in the aridity index. EcM and EcM2 indicate linear and quadratic proportions of EcM trees; AI, aridity index; soil clay, soil clay content. Lat_abs, absolute latitude.

although soil, topographical covariates, diameter threshold, and plot size all showed associations with tree species richness (Fig. 3), EcM tree proportion had a stronger association with tree species richness than all but the climatic covariates (i.e., mean annual temperature and aridity index) (Fig. 3A). In addition, the relative importance of EcM tree proportion increased from tropical to temperate to boreal forests (Fig. 3, B to D).

To further evaluate whether climate (temperature and aridity index) and soil properties (total soil nitrogen and soil pH) could influence species richness indirectly by mediating EcM tree proportion, we conducted structural equation models. We found that climate and soil properties were associated to tree species richness both directly and indirectly by affecting EcM tree proportion at the global and regional scales (Fig. 4). The relationships between EcM tree proportion (considering both linear and quadratic terms) and tree species richness ranged from unimodal to negative (Fig. 4, B to D). Compared to environmental variables, the effect of EcM tree proportion on tree species richness was stronger in tropical and boreal forests (Fig. 4, B to D), consistent with findings from random forest models. Direct effects showed that temperature was the strongest factor associated to tree species richness in boreal forests, while multiple climate and soil variables had strong effects in tropical forests (Fig. 4, B to D). In addition, temperature showed strong indirect effects on tree species richness through its influence on EcM tree proportion at the global and regional scales (Fig. 4). While the aridity index and soil pH exhibited weak indirect effects, total soil nitrogen had a strong indirect effect on tree species richness in tropical forests (Fig. 4). Both mean annual temperature and aridity index generally had positive direct effects on tree species richness at the global and regional scales (Fig. 4). However, temperature and the aridity index negatively affected EcM tree proportion, while the effect of total soil nitrogen on EcM tree proportion shifted from positive in boreal forests to negative in tropical forests (Fig. 4, B to D).

DISCUSSION

A lower tree species diversity due to EcM tree dominance in forest communities has repeatedly been hypothesized to be due to the fact that EcM fungi have stronger ability than AM fungi to weaken negative plant-soil feedbacks by specialized soil-borne pathogens (*27*). However, empirical evidence supporting the EcM dominance hypothesis is lacking, and recent regional studies in temperate forests have even produced conflicting results (*21*, *29*). By leveraging a dataset of global forest inventory plots, we found that after controlling for other environmental variables, the relationship between the proportion of EcM trees and species richness depended on latitude and aridity. Specifically, our results showed that at low latitudes and in moist conditions, this relationship was more negative, which supported the EcM dominance hypothesis. At high latitudes and in arid conditions where abiotic environments are more stressful, both EcM- and AM-dominated communities had low richness, and a mixture of the two major mycorrhizal types was associated with high tree species richness. Therefore, the findings at high latitudes

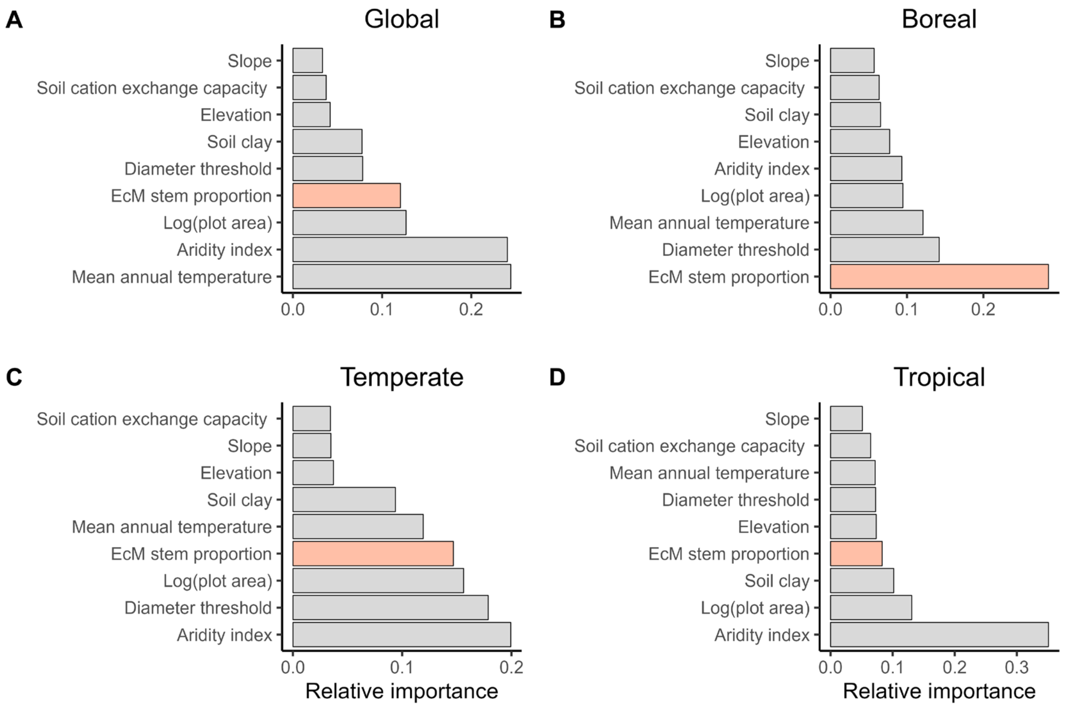

Fig. 3. The relative importance of predictors in explaining tree species richness in random forest models. (**A**) Global model ($N = 4{,}090$, $r^2 = 0.88$); (**B**) boreal model ($N = 579$, $r^2 = 0.55$); (**C**) temperate model ($N = 2{,}457$, $r^2 = 0.80$); (**D**) tropical model ($N = 1{,}054$, $r^2 = 0.75$).

and in arid conditions supported the mycorrhizal mixture hypothesis rather than the EcM dominance hypothesis. At intermediate latitudes and under intermediate aridity conditions, the relationship between tree species richness and the proportion of EcM trees was negatively unimodal, which supported the integrated hypothesis. This finding suggests that the EcM dominance hypotheses and the mycorrhizal mixture hypothesis may jointly work at intermediate latitudes and under intermediate aridity conditions, leading to a negatively unimodal relationship between tree species richness and the proportion of EcM trees in plant communities. Therefore, our results based on a global forest inventory dataset suggest that instead of favoring one of the three hypotheses proposed (Fig. 1, A to C), the mechanisms by which mycorrhizal fungi influence plant species diversity vary with environmental conditions. The importance of the EcM dominance hypothesis decreases and that of the mycorrhizal mixture hypothesis increases toward higher latitude and more arid conditions.

The variations in the relative importance of the three hypotheses along the gradients of latitudes and aridity may be due to an increasing importance of negative biotic interactions in maintaining tree species richness in forest communities as abiotic conditions become less stressful (*42*). Specifically, at low latitudes and in moist conditions, where environments are less stressful, negative plant-soil feedback and negative conspecific density dependence are relatively stronger (*38*, *39*, *44*–*46*). For example, in all three tropical biomes, EcM tree seedlings typically show a higher survival probability near their conspecific adults compared to AM tree seedlings (*16*, *47*, *48*). This stronger benefits from their conspecific adults for EcM than AM seedlings is primarily attributed to a stronger host specificity of EcM fungi (i.e., seedling survival will differ near conspecific versus heterospecific adults) and their superior physical root protection compared to AM fungi (*17*, *25*). In other words, the stronger negative biotic interactions at low latitudes and in moist conditions provide stronger potential for EcM fungi to alleviate these interactions. Therefore, the increase in the proportion of EcM trees at low latitudes and in moist conditions will substantially reduce the strength of negative plant-soil feedback, which subsequently leads to lower species richness in forest communities and a negative relationship between tree species richness and the proportion of EcM trees.

Another possible explanation of the negative relationship between tree species richness and EcM tree proportion in tropical forests is the relatively smaller species pool of EcM tree species at low latitudes compared to AM tree species (fig. S8). For example, if a tropical moist forest community is dominated by EcM trees, the species richness of this community will be low because available EcM tree species dispersing from EcM species pool is low. However, the pattern of low tree species richness in EcM-dominated communities can be formed by the following ecological processes in two stages. First, EcM trees became dominant in forest communities, which could be potentially driven by abiotic environments and/or local ecological processes such as positive plant-soil feedback. However, recent studies have found that the dominance of EcM trees in moist forests at low latitudes has a weak relationship with climate or soil conditions (*49*, *50*). Instead, positive plant-soil feedbacks of EcM tree species may be an important process that leads to their dominance in these forests (*51*, *52*). Second, EcM-dominated forest communities exhibit low tree species richness. This low species richness may be determined by the smaller species pool of EcM tree species and/or local ecological processes. Therefore, the differences in plant-soil feedbacks between EcM and AM tree species should be considered as the dominant factor in at least one of these two stages. In addition, the smaller species pool of EcM

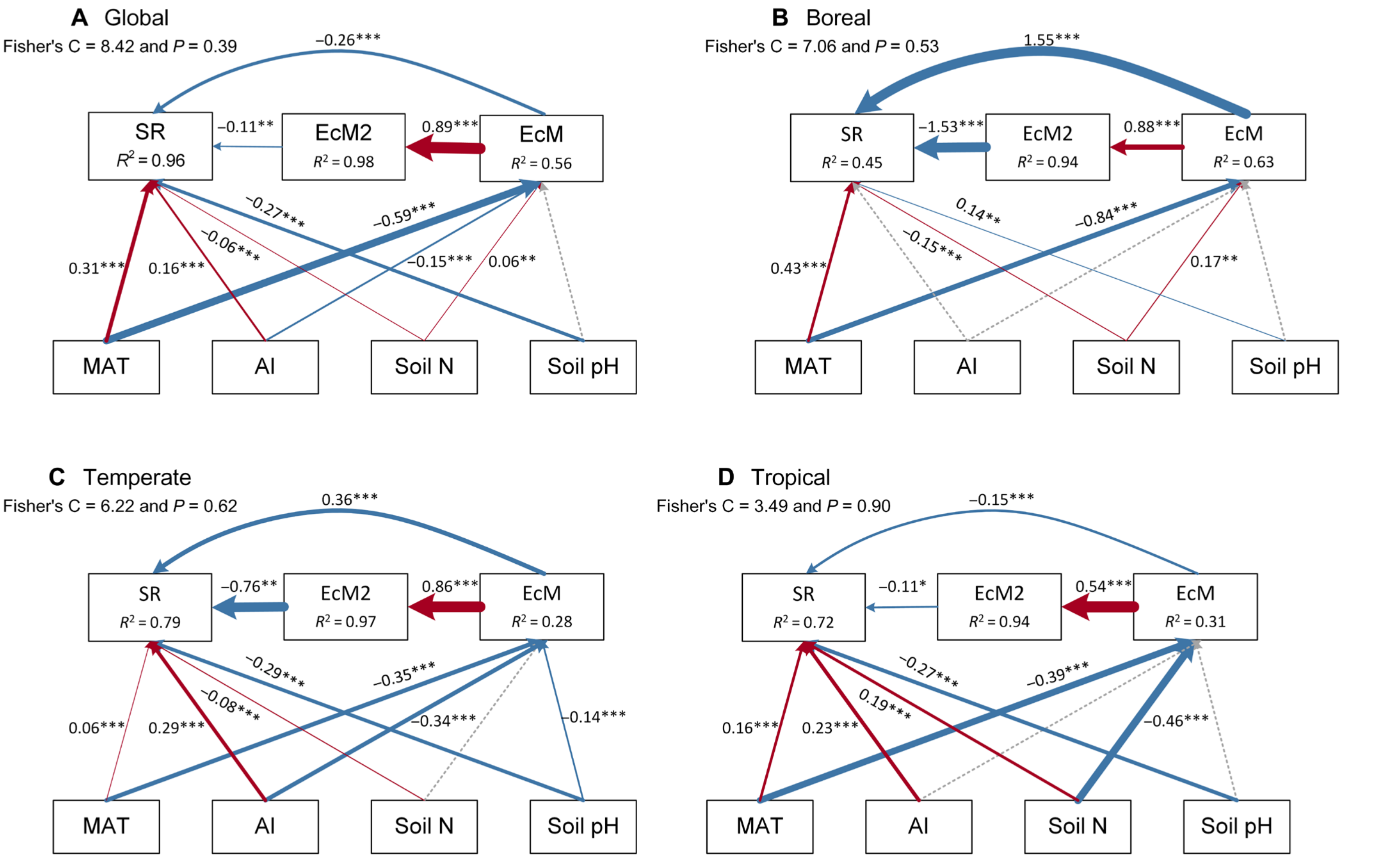

Fig. 4. Direct and indirect effects of environmental variables on species richness in forest communities. (**A**) Global model; (**B**) boreal model; (**C**) temperate model; (**D**) tropical model. The goodness of fit for structural equation models is indicated by the Fisher's C value with $P > 0.05$ suggesting good fit of models and no missing paths. Brown and blue solid lines represent significantly positive and negative relationships ($P < 0.05$), respectively. Dashed gray lines represent nonsignificant relationships ($P \geq 0.05$). Arrow width is proportional to strength if there is a significant relationship. SR, species richness; EcM and EcM2 indicate the linear and quadratic proportions of ectomycorrhizal trees; MAT, mean annual temperature; AI, aridity index; soil N, total soil nitrogen. The path from the linear to the quadratic proportion of ectomycorrhizal trees must be included in the model by piecewiseSEM package (*72*) due to their mathematical relationship (not causality). Paths linking environmental variables to EcM2 were removed because they were not significant. Including all potential paths in the models would also prevent the calculation of Fisher's C. $^*P < 0.05$, $^{**}P < 0.01$, $^{***}P < 0.001$.

than AM tree species in tropical moist forests may itself be influenced by positive plant-soil feedbacks of EcM tree species, which may then reduce local species richness. Together, the support for the EcM dominance hypothesis at low latitudes and moist conditions aligns with recent evidence from experimental and field studies (*16*, *17*, *19*, *21*–*24*). These studies, which have usually been conducted using tree seedlings, show that EcM tree species often exhibit weak negative or even positive plant-soil feedbacks and hence experience weaker conspecific negative density dependence than AM tree species in forests. Consequently, we suggest that the more negative relationship between tree species richness and the proportion of EcM trees in tropical forest communities compared with other forests is likely driven by local ecological processes (Fig. 1A), in particular, positive plant-soil feedback, rather than by a smaller species pool of EcM than of AM tree species.

With increasing latitudes and aridity, we found that the importance of the EcM dominance hypothesis declined, whereas that of the niche partitioning hypothesis enhanced. At high latitudes and in arid conditions where environments are relatively more stressful for trees, negative biotic interactions such as competition and negative plant-soil feedback of tree species may be weak, as indicated by previous studies (*42*). Instead, positive interactions and niche partitioning may be more important. Therefore, in stressful conditions, AM tree species may not have strong negative plant-soil feedbacks and negative density dependence to promote species coexistence as they do in tropical forests. In other words, because of the weak negative density dependence among AM trees in stressful environments (such as high latitudes and more arid conditions), the forest communities in these regions would have low tree species richness even when the communities are dominated by AM trees. This will result in higher tree species richness in communities with mixed mycorrhizal types than in either AM- or EcM-dominated communities. In addition, a more rapid decrease in the regional species pool of AM than EcM tree species along the latitudinal gradient may also contribute to the increased importance of mycorrhizal mixture hypothesis at high latitudes (fig. S8) and a unimodal relationship between the proportion of EcM trees and species richness. Overall, we used global data to analyze the relationship between species richness and EcM tree proportion and its variation along the gradients of latitudes and aridity index by assuming the relative importance of niche partitioning and plant-soil feedbacks. However, the numbers of forest plots in tropical regions and the Southern Hemisphere were low

compared with northern temperate regions. Incorporating more forest plots in these regions in the future would help reduce uncertainty in our analyses. Moreover, it is challenging to reveal the mechanisms underlying this relationship using inventory data. Dynamic data from repeated censuses of plant survival and recruitment or experiments could help elucidate the mechanistic relationship between species richness and EcM tree proportion.

In addition to EcM tree proportion, environmental variables, particularly climate and soil, also play a substantial role in shaping tree species richness. At low latitudes, the stronger influence of environmental variables than of EcM tree proportion is likely due to the more complex soil and climate conditions in these regions. These complex conditions may also lead to multiple environmental variables colimiting tree species richness in tropical forests, whereas boreal forests are primarily influenced by temperature (*6*). While these direct effects of climate and soil variables on tree species richness via physiological limitations have been well-documented (*6*), their indirect effects, mediated through biotic interactions (with EcM tree proportion serving as a proxy in this study), are rarely evaluated. For instance, climate (e.g., temperature and aridity index) can influence EcM tree proportion by its effects on litter decomposition (*53*, *54*). In addition, we found that effects of soil properties on EcM tree proportion, particularly total soil nitrogen, shifted from positive at high latitudes to negative at low latitudes. This latitudinal variation suggests that the role of EcM trees in biogeochemical cycling differs between low- and high-latitude forests (*54*, *55*), highlighting the ecological complexity and functional variability of EcM trees in different regions.

In summary, we used a dataset of global forest plots to test hypotheses regarding the relationships between tree mycorrhizal symbioses and tree species richness. Our analyses revealed that the relative importance of EcM dominance (i.e., the EcM dominance hypothesis) versus niche partitioning between EcM and AM species (i.e., the mycorrhizal mixture hypothesis) varied along gradients of latitude and aridity. Specifically, the EcM dominance hypothesis, which predicts a negative relationship between tree species richness and the proportion of EcM trees within communities, was more strongly supported at low latitudes and in moist conditions where strong negative biotic interactions are common. In contrast, the mycorrhizal mixture hypothesis provided a better explanation for variations in tree species richness at high latitudes and in arid conditions. We suggest that the impact of tree mycorrhizal types on the maintenance of tree species richness within forest communities depends on environmental conditions. This finding provides a global perspective on how belowground mycorrhizal fungi influence the maintenance of aboveground plant diversity and extends the classical EcM dominance hypothesis (*27*). While many other ecological and historical variables shaping global plant species diversity have been well-evaluated in previous studies (*5*), our research highlights that mutualistic interactions between plants and mycorrhizal fungi are a crucial addition to our understanding of global patterns in tree species diversity across terrestrial ecosystems.

MATERIALS AND METHODS

Forest inventory data

In this study, we assembled 442,384 ground-sourced forest inventory plots from 50 countries in five continents (fig. S1). The compilation of this comprehensive database is made possible by the Global Forest Biodiversity Initiative (GFBI) and the Science-i cyberinfrastructure. We also used an additional complementary forest inventory dataset with 1177 plots from China (*56*), 225 plots in Gentry (*57*), as well as 222 plots in SALVIAS (*58*) and 78 plots from TEAM accessed in BIEN dataset (*59*) (Fig. 1D). The final dataset consists of tree stem–level records of species identity and diameter at breast height (DBH, usually measured at 1.3 m above ground). In addition, we obtained plot-level attributes including plot ID, plot coordinates (longitude and latitude), plot size (hectare), and tree DBH threshold above which all trees in a plot were measured (centimeter). The sample intensity of forest plots is uneven across geographical regions (*53*, *60*). To eliminate the potential impacts of sample effort on our analysis, we down-sampled the forest plots in GFBI by randomly subsampling three, five, or eight plots (depending on the total sample size in these regions) within a 2° by 2° grid in North America and Europe where sample plots are most densely distributed. Our subsample consisted of 4090 plots with 579 plots in boreal forests, 2457 plots in temperate forests, and 1054 plots in tropical forests. The number of plots may vary slightly across analyses depending on the availability of environmental variables. We defined boreal forests as the regions with absolute latitude >50°, temperate forests as the regions with absolute latitude ranging between 23.5° and 50°, and tropical forests as the regions with absolute latitude <23.5° (*36*). We standardized the species names in our forest plots using the TNRS package (*61*) in R language (*62*).

Tree mycorrhizal type

We assigned the mycorrhizal type for each tree species using the FungalRoot dataset (*18*). In this dataset, each tree species is listed as EcM or "other species." The latter are mainly AM species and a low proportion of ericoid-mycorrhizal and some non-mycorrhizal species (totaling 2.6%). Initially, we categorized species by their mycorrhizal type at the genus level because mycorrhizal types are generally phylogenetically conserved within genera (*18*, *63*, *64*). Second, for the few groups in which mycorrhizal types were not consistent within a genus such as EcM-AM, we used species-level mycorrhizal types. For species classified as EcM-AM, we divided the stem number and basal area equally, attributing half to EcM trees and half to AM trees. By using the updated FungalRoot database to classify tree mycorrhizal types, we obtained the geographic pattern of EcM tree proportions, which is generally consistent with previous studies (*53*), with only minor differences. We then calculated the EcM tree proportion within each forest community using two metrics: (i) the ratio of EcM tree stem count to the total stem count and (ii) the ratio of the basal area occupied by EcM tree species to the total basal area in each plot. These two measures were used because EcM tree species on average have larger individual basal area than AM tree species; thus, the first ratio is generally smaller than the second ratio.

Covariates

To control for the effects of covariates potentially modifying the relationship between tree species richness and the EcM tree proportion, we considered 17 covariates in total, including absolute latitude, 2 climatic variables, 2 topographical variables, 11 soil variables, and 2 vegetation-survey variables. The climatic variables were mean annual temperature and aridity index, which were considered the most important variables influencing the global pattern of tree species richness in forest communities (*6*) and are associated with

the distributions of EcM trees (*53*, *55*). We used aridity index instead of precipitation because aridity index can better represent a balance between water supply and demand than precipitation. Low values of aridity index indicate dry conditions, and high values of aridity index indicate humid conditions. Mean annual temperature and aridity index were obtained from the CHELSA database and (*65*) with a spatial resolution of 30 s. Soil variables in six depths from 0 to 200 cm were obtained from the SoilGrids250m (*66*), which were aggregated in a resolution of 30 s. These variables included soil bulk density, soil cation exchange capacity, soil volumetric fraction of coarse fragments, total soil nitrogen, soil pH, the soil clay content, soil sand content, soil silt content, and soil organic carbon content. In our analyses, we used the average values of these soil variables across the six soil depth levels. Topographical variables included elevation and slope, which were extracted from the EarthEnv with a resolution of 30 s (*67*). Vegetation survey variables included DBH threshold and plot size that were obtained from original survey records. We extracted all abiotic variables from raster datasets to each plot based on longitude and latitude using the terra package (*68*) in R.

Statistical analyses

To evaluate the relationship between tree species richness and the EcM tree proportion, we used generalized linear models with tree species richness (i.e., number of tree species with DBH > DBH threshold) as the response variable and linear and quadratic terms of the EcM tree proportion and the abovementioned covariates as the predictors. We assumed a negative binomial distribution for residuals because Poisson-distributed residuals were overdispersed. To simplify the models and to avoid collinearity among the predictors, we only included the two soil variables that showed the strongest correlations with tree species richness (soil clay content and soil cation exchange capacity; see fig. S2). To account for spatial autocorrelation, we included a spatial covariate term in the model with a 2000-km neighborhood distance and weighted using inverse distance (*69*). To evaluate whether the relationship between tree species richness and the EcM tree proportion varied across the gradients of latitude and aridity conditions, we performed a model selection procedure by including interactions between EcM tree proportion, absolute latitude, and aridity index, which finally suggest a three-way interaction term in our model with the lowest Akaike information criterion (AIC) values (table S1). Because of the collinearity between absolute latitude and temperature, we excluded mean annual temperature in our model. The variance inflation factors of covariates, except for the interaction terms and the quadratic terms in our models, were all below 3.1, suggesting low collinearity among them. In addition, we also used basal area instead of stem counts for the calculation of the EcM tree proportion as independent variable and Shannon and Simpson indices of tree species diversity instead of species richness as dependent variables. The results based on these variables were presented in the Supplemental Materials. The Shannon and Simpson indices were adjusted by adding an offset of one and analyzed with generalized linear models assuming Gamma-distributed residuals.

To further assess the consistency of the observed EcM proportion–tree richness relationships across narrower environmental gradients, we also conducted a generalized linear model within each biome and ecoregion (*43*). The predictors included linear and quadratic terms of EcM tree proportion, mean annual temperature and aridity index, elevation, slope, soil clay, soil cation exchange capacity, DBH threshold, and plot size. Biome and ecoregion classifications for each plot were derived from data provided in (*43*). Notably, within this dataset, each ecoregion exhibited a more constrained variation in environmental conditions and biogeographic history that nested within biomes. For our analysis, we used a generalized linear model with Poisson-distributed residuals (or negative binomial–distributed residuals if overdispersed) for each biome and ecoregion, provided that the sample size was ≥100 plots and the range of EcM tree proportion was ≥0.5. For these separate analyses, we used all plots instead of the down-sampled dataset to retain more biomes and ecoregions (uneven sampling among separate analysis was considered unproblematic). Subsequently, to explore the potential shifts in the EcM tree proportion–tree species richness relationships along the gradients of latitude and aridity, we used Pearson correlation coefficients to examine the associations between the linear plus quadratic slopes of EcM tree proportion and absolute latitudes and aridity index.

Random forests were used to explore the relative importance of the proportion of EcM trees on tree species richness in comparison with other covariates at the global scale and in boreal, temperate, and tropical forests separately. Consistent with the generalized linear models introduced above, we included the linear term of the EcM tree proportion, as well as covariates as the predictors in these random forest models. The quadratic term of the EcM tree proportion was removed because random forests could fit nonlinear relationships well. The random forest models were calculated using the randomForest R package (*70*). We used default hyperparameters of the randomForest function in the randomForest package (*70*): number of trees = 500, number of variables randomly sampled at each split = number of predictors / 3, and relative importance of predictors = mean decrease in node impurity.

Although we anticipated that climate, soil, and the proportion of EcM trees would affect tree species richness, it was also possible that the climate and soil could influence the proportion of EcM trees within communities and hence indirectly influence tree species richness. To evaluate the direct and indirect effects of climate and soil via the proportion of EcM trees on species richness, we used structural equation modeling to assess these effects at the global scale and in boreal, temperate, and tropical forests separately. We tested how climate (represented by mean annual temperature and aridity index) and soil variables influenced tree species richness directly and indirectly by affecting the EcM tree proportion. Here, we used total soil nitrogen and soil pH to represent soil properties as previous studies have found their associations with EcM tree proportion (*55*, *71*). Since EcM tree proportion could also influence soil properties, we tested alternative structural equation models incorporating paths from EcM tree proportion to soil properties. However, our structural equation models with paths from soil properties to EcM tree proportion showed lower AIC values (table S3), indicating a better fit compared to the alternative models. Consequently, we reported results based on structural equation models with paths from soil properties to EcM tree proportion. The structural equation models were performed using the piecewiseSEM R package (*72*).

Supplementary Materials

This PDF file includes:

Figs. S1 to S8

Tables S1 to S3

REFERENCES AND NOTES

1. Y. Pan, R. A. Birdsey, J. Fang, R. Houghton, P. E. Kauppi, W. A. Kurz, O. L. Phillips, A. Shvidenko, S. L. Lewis, J. G. Canadell, P. Ciais, R. B. Jackson, S. W. Pacala, A. D. McGuire, S. Piao, A. Rautiainen, S. Sitch, D. Hayes, A large and persistent carbon sink in the world's forests. *Science* **333**, 988–993 (2011).
2. C. C. F. Boonman, J. M. Serra-Diaz, S. Hoeks, W. Y. Guo, B. J. Enquist, B. Maitner, Y. Malhi, C. Merow, R. Buitenwerf, J. C. Svenning, More than 17,000 tree species are at risk from rapid global change. *Nat. Commun.* **15**, 166 (2024).
3. W. M. Hammond, A. P. Williams, J. T. Abatzoglou, H. D. Adams, T. Klein, R. Lopez, C. Saenz-Romero, H. Hartmann, D. D. Breshears, C. D. Allen, Global field observations of tree die-off reveal hotter-drought fingerprint for earth's forests. *Nat. Commun.* **13**, 1761 (2022).
4. D. B. Lindenmayer, W. F. Laurance, J. F. Franklin, Ecology., Global decline in large old trees. *Science* **338**, 1305–1306 (2012).
5. J. H. Brown, Why are there so many species in the tropics? *J. Biogeogr.* **41**, 8–22 (2014).
6. J. Liang, J. G. P. Gamarra, N. Picard, M. Zhou, B. Pijanowski, D. F. Jacobs, P. B. Reich, T. W. Crowther, G. J. Nabuurs, S. de Miguel, J. Fang, C. W. Woodall, J. C. Svenning, T. Jucker, J. F. Bastin, S. K. Wiser, F. Slik, B. Herault, G. Alberti, G. Keppel, G. M. Hengeveld, P. L. Ibisch, C. A. Silva, H. Ter Steege, P. L. Peri, D. A. Coomes, E. B. Searle, K. von Gadow, B. Jaroszewicz, A. O. Abbasi, M. Abegg, Y. C. A. Yao, J. Aguirre-Gutierrez, A. M. A. Zambrano, J. Altman, E. Alvarez-Davila, J. G. Alvarez-Gonzalez, L. F. Alves, B. H. K. Amani, C. A. Amani, C. Ammer, B. A. Ilondea, C. Anton-Fernandez, V. Avitabile, G. A. Aymard, A. F. Azihou, J. A. Baard, T. R. Baker, R. Balazy, M. L. Bastian, R. Batumike, M. Bauters, H. Beeckman, N. M. H. Benu, R. Bitariho, P. Boeckx, J. Bogaert, F. Bongers, O. Bouriaud, P. H. S. Brancalion, S. Brandl, F. Q. Brearley, J. Briseno-Reyes, E. N. Broadbent, H. Bruelheide, E. Bulte, A. C. Catlin, R. C. Gatti, R. G. Cesar, H. Y. H. Chen, C. Chisholm, E. Cienciala, G. D. Colletta, J. J. Corral-Rivas, A. Cuchietti, A. Cuni-Sanchez, J. A. Dar, S. Dayanandan, T. de Haulleville, M. Decuyper, S. Delabye, G. Derroire, B. DeVries, J. Diisi, T. V. Do, J. Dolezal, A. Dourdain, G. P. Durrheim, N. L. E. Obiang, C. E. N. Ewango, T. J. Eyre, T. M. Fayle, L. F. N. Feunang, L. Finer, M. Fischer, J. Fridman, L. Frizzera, A. L. de Gasper, D. Gianelle, H. B. Glick, M. S. Gonzalez-Elizondo, L. Gorenstein, R. Habonayo, O. J. Hardy, D. J. Harris, A. Hector, A. Hemp, M. Herold, A. Hillers, W. Hubau, T. Ibanez, N. Imai, G. Imani, A. M. Jagodzinski, S. Janecek, V. K. Johannsen, C. A. Joly, B. Jumbam, B. Kabelong, G. A. Kahsay, V. Karminov, K. Kartawinata, J. N. Kassi, E. Kearsley, D. K. Kennard, S. Kepfer-Rojas, M. L. Khan, J. N. Kigomo, H. S. Kim, C. Klauberg, Y. Klomberg, H. Korjus, S. Kothandaraman, F. Kraxner, A. Kumar, R. Kuswandi, M. Lang, M. J. Lawes, R. V. Leite, G. Lentner, S. L. Lewis, M. B. Libalah, J. Lisingo, P. M. Lopez-Serrano, H. Lu, N. V. Lukina, A. M. Lykke, V. Maicher, B. S. Maitner, E. Marcon, A. R. Marshall, E. H. Martin, O. Martynenko, F. M. Mbayu, M. T. E. Mbuvi, J. A. Meave, C. Merow, S. Miscicki, V. S. Moreno, A. Morera, S. A. Mukul, J. C. Muller, A. Murdjoko, M. G. Nava-Miranda, L. E. Ndive, V. J. Neldner, R. V. Nevenic, L. N. Nforbelie, M. L. Ngoh, A. E. N'Guessan, M. R. Ngugi, A. S. K. Ngute, E. N. N. Njila, M. C. Nyako, T. O. Ochuodho, J. Oleksyn, A. Paquette, E. I. Parfenova, M. Park, M. Parren, N. Parthasarathy, S. Pfautsch, O. L. Phillips, M. T. F. Piedade, D. Piotto, M. Pollastrini, L. Poorter, J. R. Poulsen, A. D. Poulsen, H. Pretzsch, M. Rodeghiero, S. G. Rolim, F. Rovero, E. Rutishauser, K. Sagheb-Talebi, P. Saikia, M. N. Sainge, C. Salas-Eljatib, A. Salis, P. Schall, D. Schepaschenko, M. Scherer-Lorenzen, B. Schmid, J. Schongart, V. Seben, G. Sellan, F. Selvi, J. M. Serra-Diaz, D. Sheil, A. Z. Shvidenko, P. Sist, A. F. Souza, K. J. Sterenczak, M. J. P. Sullivan, S. Sundarapandian, M. Svoboda, M. D. Swaine, N. Targhetta, N. Tchebakova, L. A. Trethowan, R. Tropek, J. T. Mukendi, P. M. Umunay, V. A. Usoltsev, G. V. Laurin, R. Valentini, F. Valladares, F. van der Plas, D. J. Vega-Nieva, H. Verbeeck, H. Viana, A. C. Vibrans, S. A. Vieira, J. Vleminckx, C. E. Waite, H. F. Wang, E. K. Wasingya, C. Wekesa, B. Westerlund, F. Wittmann, V. Wortel, T. Zawila-Niedzwiecki, C. Zhang, X. Zhao, J. Zhu, X. Zhu, Z. X. Zhu, I. C. Zo-Bi, C. Hui, Co-limitation towards lower latitudes shapes global forest diversity gradients. *Nat. Ecol. Evol.* **6**, 1423–1437 (2022).
7. A. Esquivel-Muelbert, T. R. Baker, K. G. Dexter, S. L. Lewis, H. ter Steege, G. Lopez-Gonzalez, A. M. Mendoza, R. Brienen, T. R. Feldpausch, N. Pitman, A. Alonso, G. van der Heijden, M. Peña-Claros, M. Ahuite, M. Alexiaides, E. Á. Dávila, A. A. Murakami, L. Arroyo, M. Aulestia, H. Balslev, J. Barroso, R. Boot, A. Cano, V. C. Moscoso, J. A. Comiskey, F. Cornejo, F. Dallmeier, D. C. Daly, N. Dávila, J. F. Duivenvoorden, A. J. D. Montoya, T. Erwin, A. Di Fiore, T. Fredericksen, A. Fuentes, R. García-Villacorta, T. Gonzales, J. E. G. Andino, E. N. H. Coronado, I. Huamantupa-Chuquimaco, R. E. M. Jiménez, T. J. Killeen, Y. Malhi, C. Mendoza, H. Mogollón, P. M. Jørgensen, J. C. Montero, B. Mostacedo, W. Nauray, D. Neill, P. N. Vargas, S. Palacios, W. P. Cuenca, N. C. P. Camacho, J. Peacock, J. F. Phillips, G. Pickavance, C. A. Quesada, H. Ramírez-Angulo, Z. Restrepo, C. R. Rodriguez, M. R. Paredes, M. C. Peñuela-Mora, R. Sierra, M. Silveira, P. Stevenson, J. Stropp, J. Terborgh, M. Tirado, M. Toledo, A. Torres-Lezama, M. N. Umaña, L. E. Urrego, R. V. Martinez, L. V. Gamarra, C. I. A. Vela, E. V. Torre, V. Vos, P. von Hildebrand, C. Vriesendorp, O. Wang, K. R. Young, C. E. Zartman, O. L. Phillips, Seasonal drought limits tree species across the neotropics. *Ecography* **40**, 618–629 (2017).
8. C. M. Hulshof, M. J. Spasojevic, The edaphic control of plant diversity. *Glob. Ecol. Biogeogr.* **29**, 1634–1650 (2020).
9. J. H. Connell, On the role of natural enemies in preventing competitive exclusion in some marine animals and in rain forest trees, in *Dynamics of Populations*, P. J. den Boer, G. R. Gradwell, Eds. (Centre for Agricultural Publishing and Documentation, 1971), pp. 298–312.
10. D. H. Janzen, Herbivores and the number of tree species in tropical forests. *Am. Nat.* **104**, 501–528 (1970).
11. L. Tedersoo, M. Bahram, M. Zobel, How mycorrhizal associations drive plant population and community biology. *Science* **367**, (2020).
12. C. W. Fernandez, L. Mielke, A. Stefanski, R. Bermudez, S. E. Hobbie, R. A. Montgomery, P. B. Reich, P. G. Kennedy, Climate change-induced stress disrupts ectomycorrhizal interaction networks at the boreal-temperate ecotone. *Proc. Natl. Acad. Sci. U.S.A.* **120**, e2221619120 (2023).
13. J. Shi, X. Wang, E. Wang, Mycorrhizal symbiosis in plant growth and stress adaptation: From genes to ecosystems. *Annu. Rev. Plant Biol.* **74**, 569–607 (2023).
14. S. E. Smith, D. Read, *Mycorrhizal Symbiosis* (Academic Press, ed. 3, 2008).
15. M. Liang, X. Liu, R. S. Etienne, F. Huang, Y. Wang, S. Yu, Arbuscular mycorrhizal fungi counteract the janzen-connell effect of soil pathogens. *Ecology* **96**, 562–574 (2015).
16. M. Liang, D. Johnson, D. Burslem, S. Yu, M. Fang, J. D. Taylor, A. F. S. Taylor, T. Helgason, X. Liu, Soil fungal networks maintain local dominance of ectomycorrhizal trees. *Nat. Commun.* **11**, 2636 (2020).
17. J. A. Bennett, H. Maherali, K. O. Reinhart, Y. Lekberg, M. M. Hart, J. Klironomos, Plant-soil feedbacks and mycorrhizal type influence temperate forest population dynamics. *Science* **355**, 181–184 (2017).
18. N. A. Soudzilovskaia, S. Vaessen, M. Barcelo, J. He, S. Rahimlou, K. Abarenkov, M. C. Brundrett, S. I. F. Gomes, V. Merckx, L. Tedersoo, Fungalroot: Global online database of plant mycorrhizal associations. *New Phytol.* **227**, 955–966 (2020).
19. F. P. Teste, P. Kardol, B. L. Turner, D. A. Wardle, G. Zemunik, M. Renton, E. Laliberte, Plant-soil feedback and the maintenance of diversity in mediterranean-climate shrublands. *Science* **355**, 173–176 (2017).
20. C. S. Delavaux, J. A. LaManna, J. A. Myers, R. P. Phillips, S. Aguilar, D. Allen, A. Alonso, K. J. Anderson-Teixeira, M. E. Baker, J. L. Baltzer, P. Bissiengou, M. Bonfim, N. A. Bourg, W. Y. Brockelman, D. Burslem, L. W. Chang, Y. Chen, J. M. Chiang, C. Chu, K. Clay, S. Cordell, M. Cortese, J. den Ouden, C. Dick, S. Ediriweera, E. C. Ellis, A. Feistner, A. L. Freestone, T. Giambelluca, C. P. Giardina, G. S. Gilbert, F. He, J. Holik, R. W. Howe, W. H. Huasca, S. P. Hubbell, F. Inman, P. A. Jansen, D. J. Johnson, K. Kral, A. J. Larson, C. M. Litton, J. A. Lutz, Y. Malhi, K. McGuire, S. M. McMahon, W. J. McShea, H. Memiaghe, A. Nathalang, N. Norden, V. Novotny, M. J. O'Brien, D. A. Orwig, R. Ostertag, G. G. Parker, R. Perez, G. Reynolds, S. E. Russo, L. Sack, P. Samonil, I. F. Sun, M. E. Swanson, J. Thompson, M. Uriarte, J. Vandermeer, X. Wang, I. Ware, G. D. Weiblen, A. Wolf, S. H. Wu, J. K. Zimmerman, T. Lauber, D. S. Maynard, T. W. Crowther, C. Averill, Mycorrhizal feedbacks influence global forest structure and diversity. *Commun. Biol.* **6**, 1066 (2023).
21. F. Jiang, J. A. Lutz, Q. Guo, Z. Hao, X. Wang, G. S. Gilbert, Z. Mao, D. A. Orwig, G. G. Parker, W. Sang, Y. Liu, S. Tian, M. W. Cadotte, G. Jin, Mycorrhizal type influences plant density dependence and species richness across 15 temperate forests. *Ecology* **102**, e03259 (2021).
22. F. Jiang, K. Zhu, M. W. Cadotte, G. Jin, Tree mycorrhizal type mediates the strength of negative density dependence in temperate forests. *J. Ecol.* **108**, 2601–2610 (2020).
23. S. Jia, X. Wang, Z. Yuan, F. Lin, J. Ye, G. Lin, Z. Hao, R. Bagchi, Tree species traits affect which natural enemies drive the janzen-connell effect in a temperate forest. *Nat. Commun.* **11**, 286 (2020).
24. L. Chen, N. G. Swenson, N. Ji, X. Mi, H. Ren, L. Guo, K. Ma, Differential soil fungus accumulation and density dependence of trees in a subtropical forest. *Science* **366**, 124–128 (2019).
25. J. Davison, M. Moora, M. Öpik, A. Adholeya, L. Ainsaar, A. Bâ, S. Burla, A. G. Diedhiou, I. Hiiesalu, T. Jairus, N. C. Johnson, A. Kane, K. Koorem, M. Kochar, C. Ndiaye, M. Pärtel, Ü. Reier, Ü. Saks, R. Singh, M. Vasar, M. Zobel, Global assessment of arbuscular mycorrhizal fungus diversity reveals very low endemism. *Science* **349**, 970–973 (2015).
26. E. Laliberte, H. Lambers, T. I. Burgess, S. J. Wright, Phosphorus limitation, soil-borne pathogens and the coexistence of plant species in hyperdiverse forests and shrublands. *New Phytol.* **206**, 507–521 (2015).
27. J. H. Connell, M. D. Lowman, Low-diversity tropical rain forests: Some possible mechanisms for their existence. *Am. Nat.* **134**, 88–119 (1989).
28. S. Luo, R. P. Phillips, I. Jo, S. Fei, J. Liang, B. Schmid, N. Eisenhauer, Higher productivity in forests with mixed mycorrhizal strategies. *Nat. Commun.* **14**, 1377 (2023).
29. A. Carteron, M. Vellend, E. Laliberte, Mycorrhizal dominance reduces local tree species diversity across us forests. *Nat. Ecol. Evol.* **6**, 370–374 (2022).
30. J. M. Chase, M. A. Leibold, *Ecological niches: Linking Classical and Contemporary Aproaches* (University of Chicago Press, 2003).
31. D. Johnson, X. Liu, D. Burslem, Symbiotic control of canopy dominance in subtropical and tropical forests. *Trends Plant Sci.* **28**, 995–1003 (2023).
32. X. Liu, D. F. R. P. Burslem, J. D. Taylor, A. F. S. Taylor, E. Khoo, N. Majalap-Lee, T. Helgason, D. Johnson, J. Klironomos, Partitioning of soil phosphorus among arbuscular and ectomycorrhizal trees in tropical and subtropical forests. *Ecol. Lett.* **21**, 713–723 (2018).

33. B. D. Lindahl, A. Tunlid, Ectomycorrhizal fungi - Potential organic matter decomposers, yet not saprotrophs. *New Phytol.* **205**, 1443–1447 (2015).
34. D. J. Read, J. Perez-Moreno, Mycorrhizas and nutrient cycling in ecosystems - A journey towards relevance? *New Phytol.* **157**, 475–492 (2003).
35. C. R. See, M. Luke McCormack, S. E. Hobbie, H. Flores-Moreno, W. L. Silver, P. G. Kennedy, Global patterns in fine root decomposition: Climate, chemistry, mycorrhizal association and woodiness. *Ecol. Lett.* **22**, 946–953 (2019).
36. C. Averill, J. M. Bhatnagar, M. C. Dietze, W. D. Pearse, S. N. Kivlin, Global imprint of mycorrhizal fungi on whole-plant nutrient economics. *Proc. Natl. Acad. Sci. U.S.A.* **116**, 23163–23168 (2019).
37. I. A. Dickie, N. Koele, J. D. Blum, J. D. Gleason, M. S. McGlone, Mycorrhizas in changing ecosystems. *Botany* **92**, 149–160 (2014).
38. F. Jiang, J. A. Bennett, K. M. Crawford, J. Heinze, X. Pu, A. Luo, Z. Wang, Global patterns and drivers of plant-soil microbe interactions. *Ecol. Lett.* **27**, e14364 (2024).
39. L. S. Comita, S. A. Queenborough, S. J. Murphy, J. L. Eck, K. Xu, M. Krishnadas, N. Beckman, Y. Zhu, L. Gomez-Aparicio, Testing predictions of the janzen-connell hypothesis: A meta-analysis of experimental evidence for distance- and density-dependent seed and seedling survival. *J. Ecol.* **102**, 845–856 (2014).
40. D. W. Schemske, G. G. Mittelbach, H. V. Cornell, J. M. Sobel, K. Roy, Is there a latitudinal gradient in the importance of biotic interactions? *Annu. Rev. Ecol. Evol. Syst.* **40**, 245–269 (2009).
41. L. Sun, Y. He, M. Cao, X. Wang, X. Zhou, J. Yang, N. G. Swenson, Tree phytochemical diversity and herbivory are higher in the tropics. *Nat. Ecol. Evol.* **8**, 1426–1436 (2024).
42. R. M. Callaway, R. Brooker, P. Choler, Z. Kikvidze, C. J. Lortie, R. Michalet, L. Paolini, F. I. Pugnaire, B. Newingham, E. T. Aschehoug, C. Armas, D. Kikodze, B. J. Cook, Positive interactions among alpine plants increase with stress. *Nature* **417**, 844–848 (2002).
43. E. Dinerstein, D. Olson, A. Joshi, C. Vynne, N. D. Burgess, E. Wikramanayake, N. Hahn, S. Palminteri, P. Hedao, R. Noss, M. Hansen, H. Locke, E. C. Ellis, B. Jones, C. V. Barber, R. Hayes, C. Kormos, V. Martin, E. Crist, W. Sechrest, L. Price, J. E. M. Baillie, D. Weeden, K. Suckling, C. Davis, N. Sizer, R. Moore, D. Thau, T. Birch, P. Potapov, S. Turubanova, A. Tyukavina, N. de Souza, L. Pintea, J. C. Brito, O. A. Llewellyn, A. G. Miller, A. Patzelt, S. A. Ghazanfar, J. Timberlake, H. Kloser, Y. Shennan-Farpon, R. Kindt, J. B. Lilleso, P. van Breugel, L. Graudal, M. Voge, K. F. Al-Shammari, M. Saleem, An ecoregion-based approach to protecting half the terrestrial realm. *Bioscience* **67**, 534–545 (2017).
44. L. Hulsmann, R. A. Chisholm, L. Comita, M. D. Visser, M. de Souza Leite, S. Aguilar, K. J. Anderson-Teixeira, N. A. Bourg, W. Y. Brockelman, S. Bunyavejchewin, N. Castano, C. H. Chang-Yang, G. B. Chuyong, K. Clay, S. J. Davies, A. Duque, S. Ediriweera, C. Ewango, G. S. Gilbert, J. Holik, R. W. Howe, S. P. Hubbell, A. Itoh, D. J. Johnson, D. Kenfack, K. Kral, A. J. Larson, J. A. Lutz, J. R. Makana, Y. Malhi, S. M. McMahon, W. J. McShea, M. Mohamad, M. Nasardin, A. Nathalang, N. Norden, A. A. Oliveira, R. Parmigiani, R. Perez, R. P. Phillips, N. Pongpattananurak, I. F. Sun, M. E. Swanson, S. Tan, D. Thomas, J. Thompson, M. Uriarte, A. T. Wolf, T. L. Yao, J. K. Zimmerman, D. Zuleta, F. Hartig, Latitudinal patterns in stabilizing density dependence of forest communities. *Nature* **627**, 564–571 (2024).
45. J. A. LaManna, S. A. Mangan, A. Alonso, N. A. Bourg, W. Y. Brockelman, S. Bunyavejchewin, L. W. Chang, J. M. Chiang, G. B. Chuyong, K. Clay, R. Condit, S. Cordell, S. J. Davies, T. J. Furniss, C. P. Giardina, I. Gunatilleke, C. V. S. Gunatilleke, F. He, R. W. Howe, S. P. Hubbell, C. F. Hsieh, F. M. Inman-Narahari, D. Janik, D. J. Johnson, D. Kenfack, L. Korte, K. Kral, A. J. Larson, J. A. Lutz, S. M. McMahon, W. J. McShea, H. R. Memiaghe, A. Nathalang, V. Novotny, P. S. Ong, D. A. Orwig, R. Ostertag, G. G. Parker, R. P. Phillips, L. Sack, I. F. Sun, J. S. Tello, D. W. Thomas, B. L. Turner, D. M. Vela Diaz, T. Vrska, G. D. Weiblen, A. Wolf, S. Yap, J. A. Myers, Plant diversity increases with the strength of negative density dependence at the global scale. *Science* **356**, 1389–1392 (2017).
46. D. J. Johnson, W. T. Beaulieu, J. D. Bever, K. Clay, Conspecific negative density dependence and forest diversity. *Science* **336**, 904–907 (2012).
47. K. L. McGuire, Common ectomycorrhizal networks may maintain monodominance in a tropical rain forest. *Ecology* **88**, 567–574 (2007).
48. D. M. Newbery, I. J. Alexander, J. A. Rother, Does proximity to conspecific adults influence the establishment of ectomycorrhizal trees in rain forest? *New Phytol.* **147**, 401–409 (2000).
49. J. A. Medina-Vega, D. Zuleta, S. Aguilar, A. Alonso, P. Bissiengou, W. Y. Brockelman, S. Bunyavejchewin, D. Burslem, N. Castano, J. Chave, J. W. Dalling, A. A. de Oliveira, A. Duque, S. Ediriweera, C. E. N. Ewango, J. Filip, S. P. Hubbell, A. Itoh, S. Kiratiprayoon, S. K. Y. Lum, J. R. Makana, H. Memiaghe, D. Mitre, M. B. Mohamad, A. Nathalang, R. Nilus, N. V. Nkongolo, V. Novotny, M. J. O'Brien, R. Perez, N. Pongpattananurak, G. Reynolds, S. E. Russo, S. Tan, J. Thompson, M. Uriarte, R. Valencia, A. Vicentini, T. L. Yao, J. K. Zimmerman, S. J. Davies, Tropical tree ectomycorrhiza are distributed independently of soil nutrients. *Nat. Ecol. Evol.* **8**, 400–410 (2024).
50. K. S. H. Peh, B. Sonké, J. Lloyd, C. A. Quesada, S. L. Lewis, Soil does not explain monodominance in a central african tropical forest. *PLOS One* **6**, e16996 (2011).
51. M. Lu, L. O. Hedin, Global plant-symbiont organization and emergence of biogeochemical cycles resolved by evolution-based trait modelling. *Nat. Ecol. Evol.* **3**, 239–250 (2019).
52. A. Corrales, S. A. Mangan, B. L. Turner, J. W. Dalling, An ectomycorrhizal nitrogen economy facilitates monodominance in a neotropical forest. *Ecol. Lett.* **19**, 383–392 (2016).
53. B. S. Steidinger, T. W. Crowther, J. Liang, M. E. Van Nuland, G. D. A. Werner, P. B. Reich, G. Nabuurs, S. de Miguel, M. Zhou, N. Picard, B. Herault, X. Zhao, C. Zhang, D. Routh, K. G. Peay, GFBI consortium, Climatic controls of decomposition drive the global biogeography of forest-tree symbioses. *Nature* **569**, 404–408 (2019).
54. A. B. Keller, R. P. Phillips, Leaf litter decay rates differ between mycorrhizal groups in temperate, but not tropical, forests. *New Phytol.* **222**, 556–564 (2019).
55. M. Barceló, P. M. van Bodegom, L. Tedersoo, P. A. Olsson, N. A. Soudzilovskaia, Mycorrhizal tree impacts on topsoil biogeochemical properties in tropical forests. *J. Ecol.* **110**, 1271–1282 (2022).
56. J. Fang, Z. Shen, Z. Tang, X. Wang, Z. Wang, J. Feng, Y. Liu, X. Qiao, X. Wu, C. Zheng, Forest community survey and the structural characteristics of forests in china. *Ecography* **35**, 1059–1071 (2012).
57. A. H. Gentry, Changes in plant community diversity and floristic composition on environmental and geographical gradients. *Ann. Mo. Bot. Gard.* **75**, 1 (1988).
58. B. Enquist, B. Boyle, Salvias – the salvias vegetation inventory database. *Biodivers. Ecol.* **4**, 288–288 (2012).
59. B. S. Maitner, B. Boyle, N. Casler, R. Condit, J. Donoghue, S. M. Durán, D. Guaderrama, C. E. Hinchliff, P. M. Jørgensen, N. J. B. Kraft, B. McGill, C. Merow, N. Morueta-Holme, R. K. Peet, B. Sandel, M. Schildhauer, S. A. Smith, J.-C. Svenning, B. Thiers, C. Violle, S. Wiser, B. J. Enquist, S. McMahon, The bien r package: A tool to access the botanical information and ecology network (bien) database. *Methods Ecol. Evol.* **9**, 373–379 (2018).
60. H. Ma, T. W. Crowther, L. Mo, D. S. Maynard, S. S. Renner, J. van den Hoogen, Y. Zou, J. Liang, S. de Miguel, G.-J. Nabuurs, P. B. Reich, Ü. Niinemets, M. Abegg, Y. C. A. Yao, G. Alberti, A. M. A. Zambrano, B. V. Alvarado, E. Alvarez-Dávila, P. Alvarez-Loayza, L. F. Alves, C. Ammer, C. Antón-Fernández, A. Araujo-Murakami, L. Arroyo, V. Avitabile, G. A. Aymard, T. R. Baker, R. Bałazy, O. Banki, J. G. Barroso, M. L. Bastian, J.-F. Bastin, L. Birigazzi, P. Birnbaum, R. Bitariho, P. Boeckx, F. Bongers, O. Bouriaud, P. H. S. Brancalion, S. Brandl, F. Q. Brearley, R. Brienen, E. N. Broadbent, H. Bruelheide, F. Bussotti, R. C. Gatti, R. G. César, G. Cesljar, R. Chazdon, H. Y. H. Chen, C. Chisholm, H. Cho, E. Cienciala, C. Clark, D. Clark, G. D. Colletta, D. A. Coomes, F. C. Valverde, J. J. Corral-Rivas, P. M. Crim, J. R. Cumming, S. Dayanandan, A. L. de Gasper, M. Decuyper, G. Derroire, B. De Vries, I. Djordjevic, J. Dolezal, A. Dourdain, N. L. E. Obiang, B. J. Enquist, T. J. Eyre, A. B. Fandohan, T. M. Fayle, T. R. Feldpausch, L. V. Ferreira, L. Finér, M. Fischer, C. Fletcher, J. Fridman, L. Frizzera, J. G. P. Gamarra, D. Gianelle, H. B. Glick, D. J. Harris, A. Hector, A. Hemp, G. Hengeveld, B. Hérault, J. L. Herbohn, M. Herold, A. Hillers, E. N. H. Coronado, C. Hui, T. T. Ibanez, I. Amaral, N. Imai, A. M. Jagodziński, B. Jaroszewicz, V. K. Johannsen, C. A. Joly, T. Jucker, I. Jung, V. Karminov, K. Kartawinata, E. Kearsley, D. Kenfack, D. K. Kennard, S. Kepfer-Rojas, G. Keppel, M. L. Khan, T. J. Killeen, H. S. Kim, K. Kitayama, M. Köhl, H. Korjus, F. Kraxner, D. Kucher, D. Laarmann, M. Lang, S. L. Lewis, H. Lu, N. V. Lukina, B. S. Maitner, Y. Malhi, E. Marcon, B. S. Marimon, B. H. Marimon-Junior, A. R. Marshall, E. H. Martin, J. A. Meave, O. Melo-Cruz, C. Mendoza, C. Merow, A. M. Mendoza, V. S. Moreno, S. A. Mukul, P. Mundhenk, M. G. Nava-Miranda, D. Neill, V. J. Neldner, R. V. Nevenic, M. R. Ngugi, P. A. Niklaus, J. Oleksyn, P. Ontikov, E. Ortiz-Malavasi, Y. Pan, A. Paquette, A. Parada-Gutierrez, E. I. Parfenova, M. Park, M. Parren, N. Parthasarathy, P. L. Peri, S. Pfautsch, O. L. Phillips, N. Picard, M. T. F. Piedade, D. Piotto, N. C. A. Pitman, I. Mendoza-Polo, A. D. Poulsen, J. R. Poulsen, H. Pretzsch, F. R. Arevalo, Z. Restrepo-Correa, M. Rodeghiero, S. G. Rolim, A. Roopsind, F. Rovero, E. Rutishauser, P. Saikia, C. Salas-Eljatib, P. Saner, P. Schall, M.-J. Schelhaas, D. Schepaschenko, M. Scherer-Lorenzen, B. Schmid, J. Schöngart, E. B. Searle, V. Seben, J. M. Serra-Diaz, D. Sheil, A. Z. Shvidenko, J. E. Silva-Espejo, M. Silveira, J. Singh, P. Sist, F. Slik, B. Sonké, A. F. Souza, S. Miścicki, K. J. Stereńczak, J.-C. Svenning, M. Svoboda, B. Swanepoel, N. Targhetta, N. Tchebakova, H. T. Steege, R. Thomas, E. Tikhonova, P. M. Umunay, V. A. Usoltsev, R. Valencia, F. Valladares, F. van der Plas, T. Van Do, M. E. van Nuland, R. M. Vasquez, H. Verbeeck, H. Viana, A. C. Vibrans, S. Vieira, K. von Gadow, H.-F. Wang, J. V. Watson, G. D. A. Werner, B. Westerlund, S. K. Wiser, F. Wittmann, H. Woell, V. Wortel, R. Zagt, T. Zawiła-Niedźwiecki, C. Zhang, X. Zhao, M. Zhou, Z.-X. Zhu, I. C. Zo-Bi, C. M. Zohner, The global biogeography of tree leaf form and habit. *Nat. Plants* **9**, 1795–1809 (2023).
61. B. Boyle, N. Hopkins, Z. Lu, J. A. Raygoza Garay, D. Mozzherin, T. Rees, N. Matasci, M. L. Narro, W. H. Piel, S. J. McKay, S. Lowry, C. Freeland, R. K. Peet, B. J. Enquist, The taxonomic name resolution service: An online tool for automated standardization of plant names. *BMC Bioinformatics* **14**, 16 (2013).
62. R Core Team. 2023. R: A language and environment for statistical computing. R foundation for statistical computing, vienna, austria. https://R-project.org/.
63. M. C. Brundrett, L. Tedersoo, Evolutionary history of mycorrhizal symbioses and global host plant diversity. *New Phytol.* **220**, 1108–1115 (2018).
64. M. C. Brundrett, Mycorrhizal associations and other means of nutrition of vascular plants: Understanding the global diversity of host plants by resolving conflicting information and developing reliable means of diagnosis. *Plant Soil* **320**, 37–77 (2009).

65. R. J. Zomer, J. Xu, A. Trabucco, Version 3 of the global aridity index and potential evapotranspiration database. *Sci. Data* **9**, 409 (2022).
66. T. Hengl, J. Mendes de Jesus, G. B. Heuvelink, M. Ruiperez Gonzalez, M. Kilibarda, A. Blagotic, W. Shangguan, M. N. Wright, X. Geng, B. Bauer-Marschallinger, M. A. Guevara, R. Vargas, R. A. MacMillan, N. H. Batjes, J. G. Leenaars, E. Ribeiro, I. Wheeler, S. Mantel, B. Kempen, Soilgrids250m: Global gridded soil information based on machine learning. *PLOS ONE* **12**, e0169748 (2017).
67. G. Amatulli, S. Domisch, M. N. Tuanmu, B. Parmentier, A. Ranipeta, J. Malczyk, W. Jetz, A suite of global, cross-scale topographic variables for environmental and biodiversity modeling. *Sci. Data* **5**, 180040 (2018).
68. R. J. Hijmans. 2023 Terra: Spatial data analysis. R package version 1.7–46 https://cran.R-project.Org/package=terra.
69. B. Crase, A. C. Liedloff, B. A. Wintle, A new method for dealing with residual spatial autocorrelation in species distribution models. *Ecography* **35**, 879–888 (2012).
70. L. Breiman, Classification and regression by randomforest. *MLear* **45**, 5–32 (2001).
71. G. Zemunik, B. L. Turner, H. Lambers, E. Laliberté, Diversity of plant nutrient-acquisition strategies increases during long-term ecosystem development. *Nat. Plants* **1**, 15050 (2015).
72. J. S. Lefcheck, R. Freckleton, Piecewisesem: Piecewise structural equation modelling in r for ecology, evolution, and systematics. *Methods Ecol. Evol.* **7**, 573–579 (2015).

Acknowledgments: We are deeply grateful to all members of the Global Forest Biodiversity Initiative (GFBI), as well as the faculty and students at PKU, for efforts in field data collection and valuable data contributions to this study. Especially, we thank to N. Picard and T. Crowther for contributions in data management for GFBI. The data collection and international collaboration are facilitated by the Science-i Platform (https://science-i.org/). We thank GFBI for establishing the data standards and collaborative framework; in addition, we appreciate A. H. Gentry and the Botanical Information and Ecology Network (BIEN) for sharing the inventory data. We also acknowledge the efforts of the editors and the valuable suggestions and comments from the reviewers. This work is dedicated in memory of K.K. **Funding:** This study was supported by the National Natural Science Foundation of China (#32125026 and #32301329) and the National Key Research Development Program of China (#2022YFF0802300). The Science-i cyberinfrastructure is supported in parts by the National Science Foundation of the United States (award # 2311762) and the Department of Forestry and Natural Resources, Purdue University. F.J. was funded by the National Natural Science Foundation of China (#32201300). J.L. was supported by grants from the Purdue University, Office of Agricultural Research and Graduate Education and Executive Vice President for Research through their joint Elevating the Visibility of Research initiative. A.F.S. acknowledges that this study used GFBI plot data originally collected in Brazil with funding by Conselho Nacional de Desenvolvimento Científico e Tecnológico (CNPq) (project 520053/1998-2). We thank the team from Mexico for generating field data through the Monitoreo Nacional Forestal (MONAFOR) network with funding from the Comisión Nacional Forestal (CONAFOR) and local projects supported by ejidos and communities. S.d.-M. benefited from a Serra-Húnter fellowship provided by the Government of Catalonia (Generalitat de Catalunya), and his research was supported by the project with grant number PID2022-139558OB-I00 funded by MCIN/AEI and by "ERDF A way of making Europe", and by the project with grant number PCI2023-146021-2 funded by MCIN/AEI and by the European Union. J.A.-G. was funded by the Natural Environment Research Council (NERC; NE/T011084/1) and the Oxford University John Fell Fund (10667). A.C. acknowledges the project funded under the National Recovery and Resilience Plan (NRRP), Mission 4 Component 2 Investment 1.4 - Call for tender no. 3138 of 16 December 2021, rectified by decree n.3175 of 18 December 2021 of Italian Ministry of University and Research funded by the European Union–NextGenerationEU under award number: Project code CN_00000033, concession decree no. 1034 of 17 June 2022 adopted by the Italian Ministry of University and Research, CUP B83C22002930006, Project title "National Biodiversity Future Centre - NBFC". We are also grateful to all the ministries and agencies from the Government of Spain, and to all the people and institutions who supported the collection, compilation, and coordination of forest inventory data, also including the Spanish National Forest Inventories. J.A. and J.D. were supported by research grants 23-05272S and 25-15727S (J.A.) of the Czech Science Foundation, Czech-Estonian Academy of Science mobility grant EAS-EE-CZ-24-2 (J.D.), and the long-term research development project no. RVO 67985939 of the Czech Academy of Sciences. P.B.R. was supported by the US National Science Foundation Biological Integration Institutes grant NSF-DBI-2021898. C.A.J. is thankful to the State of São Paulo Research Foundation (FAPESP) through the BIOTA/FAPESP Program Grants 2003/12595-7; 2010/20811-7, 2012/51872-5 and to the Brazilian National Research Council (CNPq) PELD Program grant CNPq 403710/20120). L.M. and L.K. acknowledge that plot data from Central Siberian forests were collected with support from the State Assignment of the Ministry of Science and Higher Education of the Russian Federation [L.M. was supported by project no. FWES-2024-0023 (state registration number 124012900558-7), and L.K. was supported by project no. FWES-2024-0028 (state registration number 124012900557-0)]. G.V.L. acknowledges the EU, the Next Generation EU Mission4 "Education and Research,",Component 2: "From research to business" Investment 3.I: "Fund for the realization of an integrated system of research andinnovation infrastructures", Project IR0000032 Italian Integrated Environmental Research Infrastructures System (ITINERIS) (CUP B53C22002150006). K.S. acknowledges Project LIFE+ForBioSensing (contract number LIFE13ENV/PL/000048) and Poland's National Fund for Environmental Protection and Water Management (contract number 485/2014/WN10/OPNMLF/D) and project "AFTER FBS – maintenance of ForBioSensing project performance indicators," which is funded by the Forest Research Institute's own research fund (no. 261509). J.A.D would like to acknowledge the Science and Engineering Research Board, Department of Science and Technology, Govt. of India for funding (ref. no.: SRG/2022/002286) and SRM University - AP for SEED Grant (SRMAP/URG/E&PP/2022–23/12). H.Y.H.C. acknowledges the Natural Sciences and Engineering Research Council of Canada (RGPIN-2019-05109, RTI-2017-00358, and STPGP506284) and the Canadian Foundation for Innovation (36014). S.K. acknowledges the Science and Engineering Research Board, Department of Science and Technology (DST-SERB) for funding (ref. no.: PDF/2021/003742/LS). J.D. was supported by the Czech Science Foundation (GACR 24-11954S) and by the Ministry of Education, Youth, and Sport of the Czech Republic (MSMT) (#VES24, INTER-ACTION, LUAUS24258). H.L. was funded by the National Natural Science Foundation of China (31800374). Z.T. was funded by the National Natural Science Foundation of China (32025025). I.D. was funded by Ministry of Science, Technological Development and Innovation of Serbia (451-03-66/2024-03/200027). K.A.K. was funded by Czech Science Foundation (23-05272S), Long-term research development project of the Czech Academy of Sciences (RVO 67985939), and Scientific framework of the Botanical Garden-Institute FEB RAS (FWFR-2022-0008) (122040800089-2). P.B. was funded by BOF, Ghent University (Methusalem). M.Sv. was funded by Czech Science Foundation (24-12210 K). J.J.C.-R. was funded by Comisión Nacional Forestal (CONAFOR-115900). G.-J.N. was funded by HEurope SUPERB (101036849), HEurope Monifun (101134991), Europe Wildcard (101081177), and Dutch Mnistry Agriculture, Food Security, Fisheries and Nature. C.A.S. acknowledges the NASA's grants (ICESat-2, 80NSSC23K0941), Carbon Monitoring System (CMS, grant 80NSSC23K1257), and Commercial Smallsat Data Scientific Analysis (CSDSA, grant 80NSSC24K0055). M.Ba. acknowledges the BOS Foundation, the Indonesian Institute of Sciences (LIPI), the Direktorat Fasilitasi Organisasi Politik dan Kemasyarakatan, Departamen Dalam Negri, and the BKSDA Palangkaraya for permission to work in the MAWAS region of Indonesia. She was supported by The American Society of Primatologists, the Duke University Graduate School, the L.S.B. Leakey Foundation, the National Science Foundation (grant no. 0452995), and the Wenner-Gren Foundation for Anthropological Research (grant no. 7330). J.S. acknowledges the Brazilian National Research Council (CNPq) and State of Amazonas Research Foundation (FAPEAM) PELD Program grants (CNPq 441811/2020-5 and FAPEAM 01.02.016301.02630/2022-76). E.C. acknowledges funding from AdAgriF—Advanced methods of greenhouse gases emission reduction and sequestration in agriculture and forest landscape for climate change mitigation (CZ.02.01.01/00/22_008/0004635). A.B. was supported by state funding contract 'Biodiversity and Ecosystem Functions of Forests' (124013000750-1). **Author contributions:** Conceptualization: F.J., X.P., Z.W., and J.L. Methodology: F.J., X.P., Z.W., J.L., B.S., and P.B.R. Formal analysis: F.J. and X.P. Data curator: F.J., X.P., B.S., P.B.R., J.L., A.O.A., J.A.-G., A.M.A.Z., J.A., J.G.Á.-G., L.F.A., B.H.K.A., C.A., G.A.A., N.B.K., M.L. Ba., J.F.-B., M. Bau., P.B., S.N.B., A.B., F.Q.B., S.B., J.B.-R., E.N.B., G.Č., H.Y.H.C., C.C., W.C., E.C., C.J.C., A.C., J.J.C.-R., J.A.D., S.D., S.d.-M., A.A.D., G.D., I.D., T.V.D., J.D., A.D., T.E., A.B.F., L.F., R.C.G., D.G., M.S.G.E., E.G., D.J.H., A.H., B.H., C.H., N.I., A.M.J., C.J., C.A.J., V.N.K., K.K., J.N.K., E.K., G.K., M.L.K., C.K., K.A.K., S.K., F.K., L.K., D.K., A. Ku., A. Kv., G.V.L., R.V.L., M.B.L., E.S.L., H.L., S.L., Y.L., E.M., A.R.M., R.V.M., R.M., W.M., A.N., M.G.N.-M., J.N., A.M.M., S.M., T.M., L.M., S.A.M., G.-J.N., V.J.N., R.N., A.E.N., M.N., A.P., E.I.P., M.P., N.P., P.L.P., S.P., M.T.F.P., G.P., A.D.P., J.R.P., H.P., M.R., E.R., P. Sai., P. San., D. Sc., J.S., E.B.S., D. Sh., Z.S., S. Sh., A.S., C.A.S., P. Si., F.S., W.S., A.F.S., K.S., S. Su., M. Svá., M. Svo., Z.T., N. Ta., N. Tc., E.T., L.T., D.J.V.-N., H.V., S.A.V., C.V., A.S.V., F.K.V., H.F.W., S.W., X.W., F.W., C.Z., B.Z., I.C.Z.-B., J.F., and Z.W. Writing—original draft: F.J. and X.P. Writing—review and editing: F.J., X.P., B.S., P.B.R., J.L., A.O.A., J.A.-G., A.M.A.Z., J.A., J.G.Á.-G., L.F.A., B.H.K.A., C.A., G.A.A., N.B.K., M.L. Ba., J.F.-B., M. Bau., P.B., S.N.B., A.B., F.Q.B., S.B., J.B.-R., E.N.B., G.Č., H.Y.H.C., C.C., W.C., E.C., C.J.C., A.C., J.J.C.-R., J.A.D., S.D., S.d.-M., A.A.D., G.D., I.D., T.V.D., J.D., A.D., T.E., A.B.F., L.F., R.C.G., D.G., M.S.G.E., E.G., D.J.H., A.H., B.H., C.H., N.I., A.M.J., C.J., L.J., C.A.J., V.N.K., K.K., J.N.K., E.K., G.K., M.L.K., C.K., K.A.K., S.K., F.K., L.K., D.K., A. Ku., A. Kv., G.V.L., R.V.L., M.B.L., E.S.L., H.L., S.L., Y.L., E.M., A.R.M., R.V.M., R.M., W.M., A.N., M.G.N.-M., J.N., A.M.M., S.M., T.M., L.M., S.A.M., G.-J.N., V.J.N., R.N., A.E.N., M.N., A.P., E.I.P., M.P., N.P., P.L.P., S.P., M.T.F.P., G.P., A.D.P., J.R.P., H.P., M.R., E.R., P. Sai., P. San., D. Sc., J.S., E.B.S., D. Sh., Z.S., S. Sh., A.S., C.A.S., P. Si., F.S., W.S., A.F.S., K.S., S. Su., M. Svá., M. Svo., Z.T., N. Ta., N. Tc., E.T., L.T., D.J.V.-N., H.V., S.A.V., C.V., A.S.V., F.K.V., H.F.W., S.W., X.W., F.W., C.Z., B.Z., I.C.Z.-B., J.F., and Z.W. Project administration: Z.W. and J.L. **Competing interests:** The authors declare that they have no competing interests. **Data and materials availability:** All data needed to evaluate the conclusions in the paper are present in the paper and/or the Supplementary Materials. Original data used in the Global Forest Biodiversity Initiative (GFBI) are subject to restrictions set by the plot data owners. As a result, researchers interested in accessing the data are required to submit data requests through the GFBI platform via Science-i (https://science-i.org/). Access to the data will be granted upon approval from the respective data contributors.

Submitted 2 October 2024
Accepted 12 May 2025
Published 13 June 2025
10.1126/sciadv.adt5743

[1]Institute of Ecology and State Key Laboratory for Vegetation Structure, Function and Construction (VegLab), College of Urban and Environmental Sciences, Peking University, Beijing, China. [2]Remote Sensing Laboratories, Department of Geography, University of Zürich, Zürich, Switzerland. [3]Department of Forest Resources, University of Minnesota, St Paul, MN 55108, USA. [4]Hawkesbury Institute for the Environment, Western Sydney University, Penrith, New South Wales, Australia. [5]Institute for Global Change Biology and School for Environment and Sustainability, University of Michigan, Ann Arbor, MI 48109, USA. [6]Forest Advanced Computing and Artificial Intelligence Laboratory (FACAI), Department of Forestry and Natural Resources, Purdue University, West Lafayette, IN, USA. [7]Environmental Change Institute, School of Geography and the Environment, University of Oxford, Oxford, UK. [8]Leverhulme Centre for Nature Recovery, University of Oxford, Oxford, UK, OX13QY. [9]Spatial Ecology and Conservation (SPEC) Lab, Center for Latin American Studies, University of Florida, Gainesville, FL 32611 USA. [10]Institute of Botany of the Czech Academy of Sciences, Třeboň, Czech Republic. [11]Faculty of Forestry and Wood Sciences, Czech University of Life Sciences, Prague, Czech Republic. [12]Departamento de Ingeniería Agroforestal, Universidad de Santiago de Compostela, Lugo, Spain. [13]Center for Tropical Research, Institute of the Environment and Sustainability, University of California, Los Angeles, Los Angeles, CA 90095, USA. [14]Université Jean Lorougnon Guédé, Unité de Formation et de Recherche en Agroforesterie, BP 150, Daloa, Ivory Coast. [15]Université Nangui Abrogoua, Unité de Formation et de Recherche en Sciences de la Nature, 02, BP 801 Abidjan 02, Laboratoire d'Écologie et du Développement durable (LEDD), Abidjian, Ivory Coast. [16]Silviculture and Forest Ecology, Georg August-University of Göttingen, Göttingen, Germany. [17]UNELLEZ-Guanare, Programa de Ciencias del Agro y el Mar, Herbario Universitario (PORT), Portuguesa, Venezuela. [18]Jardín Botánico de Bogotá José Celestino Mutis, Cl. 63 #68-95, Bogotá DC., Colombia. [19]Department of Ecology, French Institute of Pondicherry, Puducherry 605001, India. [20]Proceedings of the National Academy of Sciences, Washington, DC 20001, USA. [21]Department of Evolutionary Anthropology, Duke University, Durham, NC 27710, USA. [22]TERRA Teaching and Research Centre, Gembloux Agro Bio-Tech, Université de Liège, Gembloux, Belgium. [23]Q-ForestLab, Department of Environment, Ghent University, Gent, Belgium. [24]Isotope Bioscience Laboratory - ISOFYS, Department of Green Chemistry and Technology, Ghent University, Gent, Belgium. [25]Sikhote-Alin State Nature Biosphere Reserve, Terney, Russia. [26]Isaev Center for Forest Ecology and Productivity of the Russian Academy of Sciences, Moscow, Russia. [27]Department of Natural Sciences, Manchester Metropolitan University, Chester Street, Manchester M1 5GD, UK. [28]Vascular Surgery, Newcastle University, Newcastle, UK. [29]Facultad de Ciencias Forestales y Ambientales, Universidad Juárez del Estado de Durango, Durango, Mexico. [30]Spatial Ecology and Conservation Laboratory, School of Forest, Fisheries, and Geomatics Sciences, University of Florida, Gainesville, FL 32611, USA. [31]Department of Spatial Regulation, GIS and Forest Policy, Institute of Forestry, Belgrade, Serbia. [32]Faculty of Natural Resources Management, Lakehead University, Thunder Bay, Ontario, Canada. [33]Institute for Global Change Biology, School for Environment and Sustainability, University of Michigan, Ann Arbor, MI 48109, USA. [34]Institute of Integrative Biology, ETH Zurich (Swiss Federal Institute of Technology), Zurich, Switzerland. [35]IFER – Institute of Forest Ecosystem Research, Jilove u Prahy, Czech Republic. [36]Global Change Research Institute of the CAS, Brno, Czech Republic. [37]Nicholas School of the Environment, Duke University, Durham, NC 27710, USA. [38]Forest Modelling Lab., National Research Council of Italy, Institute for Agriculture and Forestry Systems in the Mediterranean, (CNR-ISAFOM), Perugia, Italy. [39]National Biodiversity Future Center (NBFC), Palermo, Italy. [40]TEaM (Terrestrial Ecology and Modelling) Lab, Department of Environmental Science and Engineering, School of Engineering and Sciences, SRM University-AP, Amaravati 522240, India. [41]Centre for Geospatial Technology, SRM University-AP, Amaravati 522240, India. [42]Centre for Structural and Functional Genomics & Quebec Centre for Biodiversity Science, Biology Department, Concordia University, Montreal, Quebec, Canada. [43]Department of Agricultural and Forest Sciences and Engineering, University of Lleida, Lleida, Spain. [44]Forest Science and Technology Centre of Catalonia (CTFC), Solsona, Spain. [45]Department of Ecology and Environmental Sciences, School of Life Sciences, Pondicherry University, Puducherry 605014, India. [46]Cirad, UMR EcoFoG (AgroParisTech, CNRS, INRAE, Université des Antilles, Université de Guyane), Kourou, French Guiana. [47]Institute of Forestry, Beograd, Serbia. [48]Department of Silviculture Foundation, Silviculture Research Institute, Vietnamese Academy of Forest Sciences, Hanoi, Vietnam. [49]Faculty of Sciences, University of South Bohemia, Ceske Budejovice, Czech Republic. [50]Queensland Herbarium and Biodiversity Science, Department of Environment, Tourism, Science and Innovation, Toowong, QLD, Australia. [51]Ecole de Foresterie Tropicale, Université Nationale d'Agriculture, Kétou, Benin. [52]Research and Innovation Centre, Fondazione Edmund Mach, via E. Mach 1, 38098 San Michele all'Adige, Trento, Italy. [53]Department of Biological, Geological, and Environmental Sciences (BiGeA), University of Bologna, Bologna, Italy. [54]Instituto Politécnico Nacional, Centro Interdisciplinario de Investigación para el Desarrollo Integral Regional, Durango, México. [55]Royal Botanic Garden Edinburgh, Edinburgh, UK. [56]Department of Plant Sciences, University of Oxford, Oxford OX13RB, UK. [57]CIRAD, UPR Forêts et Sociétés, F-34398 Montpellier, France. [58]Forêts et Sociétés, Univ Montpellier, CIRAD, Montpellier, France. [59]Centre for Invasion Biology, Department of Mathematical Sciences, Stellenbosch University, Stellenbosch 7602, South Africa. [60]National Institute for Theoretical and Computational Sciences (NITheCS), African Institute for Mathematical Sciences, Cape Town 7945, South Africa. [61]Department of Forest Science, Tokyo University of Agriculture, Tokyo, Japan. [62]Institute of Dendrology, Polish Academy of Sciences, Parkowa 5, 62-035 Kórnik, Poland. [63]Poznań University of Life Sciences, Department of Game Management and Forest Protection, Wojska Polskiego 71D, 60-625 Poznań, Poland. [64]School of Biological Sciences, Georgia Institute of Technology, Atlanta, GA, USA. [65]Department of Plant Biology, Institute of Biology, University of Campinas, UNICAMP, Campinas, Brazil. [66]Brazilian Platform for Biodiversity and Ecosystem Services/BPBES, Campinas/SP, Brazil. [67]Forestry Faculty, Mytischi Branch of Bauman Moscow State Technical University, 1st Institutskaya street, 1, 141005, Mytishchi, Moscow, Russia. [68]Integrative Research Center, Field Museum, Chicago, IL 60605, USA. [69]Labo Botanique, Université Félix Houphouët-Boigny, Abidjan, Ivory Coast. [70]UniSA STEM and Future Industries Institute, University of South Australia, Adelaide, South Australia, Australia. [71]Department of Botany, Dr. Harisingh Gour Vishwavidyalaya (A Central University), Sagar - 470003, Madhya Pradesh, India. [72]School of Forest, Fisheries, and Geomatics Sciences, University of Florida, Gainesville, FL 32611, USA. [73]Botanical Garden-Institute FEB RAS, Vladivostok, Russia. [74]Research Group on Agriculture, Forestry, and Ecosystem Services (AFE), International Institute for Applied Systems Analysis, Laxenburg, A-2361, Austria. [75]V. N. Sukachev Institute of Forest, Federal Research Center, Krasnoyarsk Science Center of the Siberian Branch of the Russian Academy of Sciences, Krasnoyarsk, Russia. [76]Department of Environmental Management, Institute of Environmental Engineering, RUDN University, 6 Miklukho-Maklaya St, Moscow 117198, Russia. [77]Department of Geography, Institute of Science, Banaras Hindu University, Varanasi 221005, India. [78]Department of Forestry and Natural Resources, Purdue University, West Lafayette, IN 47907, USA. [79]Denezhkin Kamen Zapovednik, Sverdlovskaya Oblast, Severouralskiy Rain. Vsevolodo-Blagodatskoe, Russia. [80]Research Institute on Terrestrial Ecosystems, National Research Council, Montelibretti Research Area, Rome, Italy. [81]Department of Forest Engineering, Federal University of Viçosa (UFV), Viçosa, Brazil. [82]NASA Postdoctoral Program Fellow, Goddard Space Flight Center, Greenbelt, MD 20771, USA. [83]Department of Plant Biology, Faculty of Science, University of Yaoundé I, Yaoundé, Cameroon. [84]Plant Systematics and Ecology Laboratory (LaBosystE), Higher Teacher's Training College, University of Yaoundé I, Yaoundé, Cameroon. [85]State nature reserve 'Bastak,' 69a Sholom-Aleichem St., 69a, Birobidzhan, Russia. [86]Faculty of Forestry, Qingdao Agricultural University, Qingdao, China. [87]German Centre for Integrative Biodiversity Research (iDiv) Halle-Jena-Leipzig, Leipzig, Germany. [88]Department of Evolution, Ecology, and Behaviour, University of Liverpool, Crown street, Liverpool L69 7BE, UK. [89]University of the Sunshine Coast, Sippy Downs, QLD 4556, Australia. [90]Flamingo Land Ltd., North Yorkshire YO17 6UX, UK. [91]Forest Research Institute, University of the Sunshine Coast, Sippy Downs, QLD 4655, Australia. [92]Jardín Botánico de Missouri, St. Louis, MO 63110, USA. [93]Faculty of Forestry and Wood Sciences, Czech University of Life Sciences Prague, Kamýcká 129, 165 00 Prague, Czech Republic. [94]Queensland Herbarium, Toowong, QLD, Australia. [95]Escuela Politécnica Superior de Ingeniería, Campus Terra, Universidad de Santiago de Compostela, Lugo, España. [96]Colegio de Ciencias y Humanidades, Universidad Juárez del Estado de Durango, Dgo, México. [97]Universidad Nacional de San Antonio Abad del Cusco, Cusco, Peru. [98]Department of Forest Management Planning, Dendrometry and Forest Economics, Faculty of Forestry, Warsaw University of Life Sciences – SGGW, Nowoursynowska 159 St., 02-776 Warsaw, Poland. [99]Mountain-Taiga research station, Federal Scientific Center of the East Asia Terrestrial Biodiversity, Far Eastern Branch of the Russian Academy of Sciences, Gorno-Tayozhnoye, Primorsky Krai 692533, Russia. [100]Department of Environment and Development Studies, United International University, Dhaka 1212, Bangladesh. [101]Tropical Forests and People Research Centre, University of the Sunshine Coast, Maroochydore DC, QLD, Australia. [102]Wageningen Environmental Research, Wageningen University & Research, Wageningen, Netherlands. [103]Forest Ecology and Forest Management Group, Wageningen University & Research, Wageningen, Netherlands. [104]Institute of Forestry, Belgrade, Serbia. [105]UFR Biosciences, University Félix Houphouët-Boigny, Abidjan, Ivory Coast. [106]Centre for Forest Research, Université du Québec à Montréal, Montréal, Québec, Canada. [107]Instituto Nacional de Tecnología Agropecuaria (INTA), EEA Santa Cruz, Río Gallegos, Santa Cruz, Argentina. [108]Urban Management and Planning, School of Social Sciences, Western Sydney University, Penrith, New South Wales, Australia. [109]Monitoring and Sustainable Use of Wetlands (MAUA), National Institute for Amazonian Research - INPA, Av. Andr´e Araújo, 2.936 - Petr´opolis, Manaus CEP 69067-375, Amazonas, Brazil. [110]The Nature Conservancy, 2424 Spruce St., Boulder, CO 80302, USA. [111]Chair for Forest Growth and Yield Science, TUM School for Life Sciences, Technical University of Munich, Munich, Germany. [112]Info Flora, Geneva, Switzerland. [113]Department of Botany, Banaras Hindu University, Varanasi 221005, India. [114]datascientist.ch, Wallisellen, Switzerland. [115]Instituto Nacional de Pesquisas da Amazônia—INPA, Grupo Ecologia. Monitoramento e Uso Sustentável de Áreas Úmidas MAUA, Manaus, Brazil. [116]Department of Biology, Concordia University, 7141 Sherbrooke Street West, Montreal, Quebec H4B 1R6, Canada. [117]Department of Biology, McGill University, 1205 Avenue Docteur Penfield, Montreal, Quebec H3A 1B1, Canada. [118]Forest Biometrics and Remote Sensing Laboratory (Silva Lab), School of Forest,

Fisheries, and Geomatics Sciences, University of Florida, Gainesville, FL 32611, USA. [119]Cirad, Forests & Societies, University of Montpellier, Montpellier, France. [120]Environmental and Life Sciences, Faculty of Science, Universiti Brunei Darussalam, Bandar Seri Begawan, Brunei Darussalam. [121]Departamento de Ecologia, Universidade Federal do Rio Grande do Norte (UFRN), Natal, RN, Brazil. [122]Forest Research Institute, Department of Geomatics, Braci Leśnej 3 Street, Sękocin Stary, 05-090 Raszyn, Poland. [123]Department of Forest Botany, Dendrology and Geobiocoenology, Mendel University in Brno, Brno, Czech Republic. [124]Center for Forest Ecology and Productivity of the Russian Academy of Science, Moscow, Russia. [125]Royal Botanic Gardens Kew, London, UK. [126]Environmental Studies and Research Center, Universidade Estadual de Campinas, UNICAMP, Campinas, Brazil. [127]Department of Physical Geography and Ecosystem Science, Lund University, Lund, Sweden. [128]Siberian Federal University, Krasnoyarsk, Russia. [129]Forest Research Institute of the Karelian Research Centre of the Russian Academy of Sciences, Republic of Karelia, 11 Ul. Pushkinskaya, Petrozavodsk 185910, Russia. [130]Sanya Nanfan Research Institute of Hainan University, Hainan Yazhou Bay Seed Laboratory, Hainan University, Sanya 572025, China. [131]School of Ecology and Nature Conservation, Beijing Forestry University, Beijing 100083, China. [132]Department of Wetland Ecology, Institute for Geography and Geoecology, Karlsruhe Institute for Technology, Karlsruhe, Germany. [133]Ecole Supérieure d'Agronomie, Institut National Polytechnique Félix Houphouët-Boigny (ESA/INP-HB), BP 1093, Yamoussoukro, Côte d'Ivoire. [134]College of Ecology and Environmental Sciences, Yunnan University, Chenggong, Kunming 650500, China.
*Corresponding author. Email: zhiheng.wang@pku.edu.cn (Z.W.); albeca.liang@gmail.com (J.L.)
†These authors contributed equally to this work.
‡Deceased.
