# Supplementary material for: Mycorrhizal symbioses and tree diversity in global forest communities
Source: Sci Adv. 2025 Jun 13;11(24):eadt5743. doi: 10.1126/sciadv.adt5743 (PMC12164979; doi:10.1126/sciadv.adt5743)

Science Advances
AAAS

Supplementary Materials for

Mycorrhizal symbioses and tree diversity in global forest communities

Feng Jiang *et al.*

Corresponding author: Zhiheng Wang, zhiheng.wang@pku.edu.cn; Jingjing Liang, albeca.liang@gmail.com

Sci. Adv. **11**, eadt5743 (2025)
DOI: 10.1126/sciadv.adt5743

This PDF file includes:

Figs. S1 to S8
Tables S1 to S3

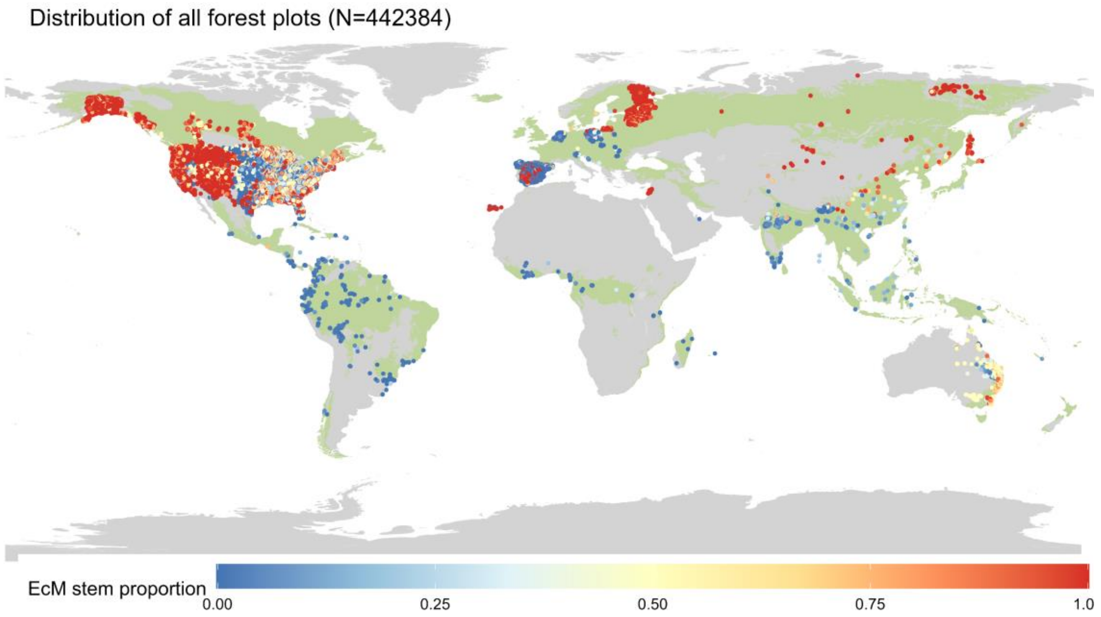

Fig. S1. Distribution of global all forest plots and proportion of EcM tree stems. This full dataset is used for analyses within biomes and ecoregions.

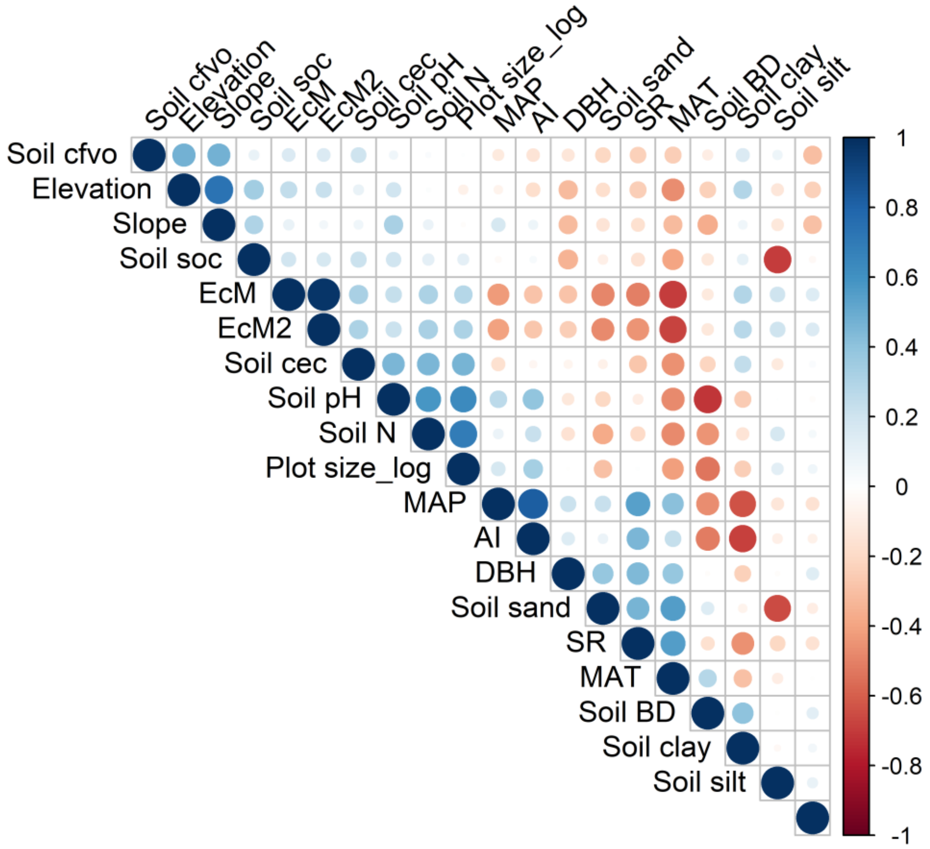

Fig. S2. Correlation matrix among species richness (SR), climate, soil, topography, and survey variables. Climate: MAT, mean annual temperature; MAP, mean annual precipitation; AI, aridity index. Soil: soil clay, soil clay content; soil cec, soil cation exchange capacity; soil cfvo, soil volumetric fraction of coarse fragments; soil soc, soil organic carbon content; soil sand, soil sand content; soil silt, soil silt content; soil N, total soil nitrogen; soil BD, soil bulk density. Topography: elevation, slope. Survey information: DBH, diameter at breast height threshold in plot survey. EcM, linear proportion of EcM stems; EcM2, quadratic proportion of EcM stems.

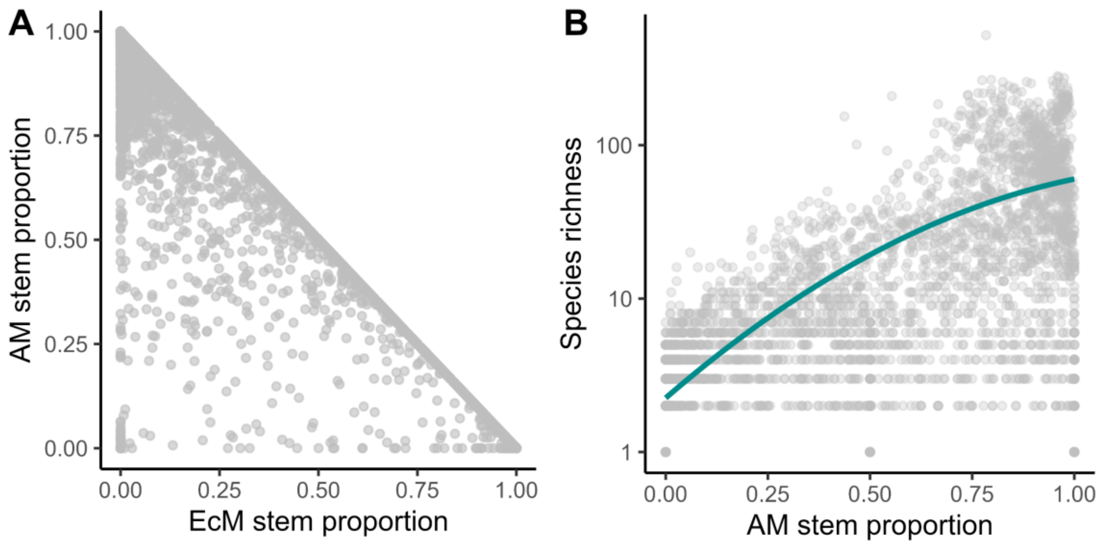

Fig. S3. Evaluation of the association with species richness using AM stem proportion. A, the relationship between proportions of AM and EcM tree stems; **B,** the relationship between AM stem proportion and species richness without controlling for the covariates.

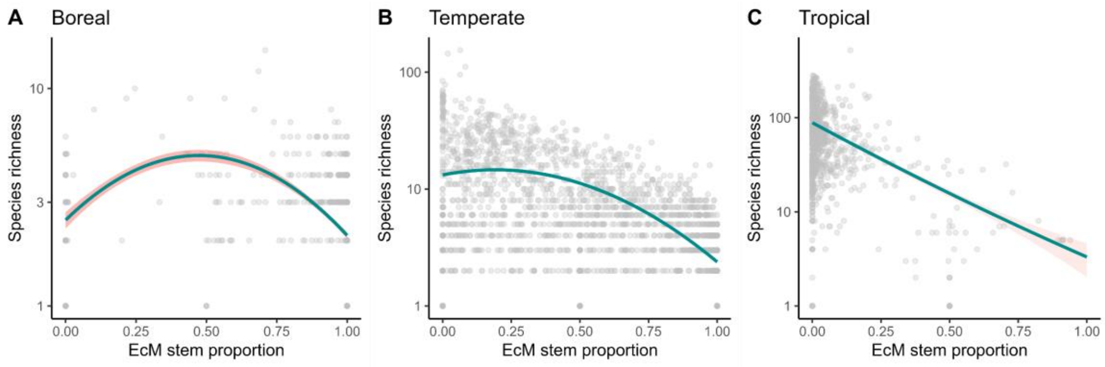

Fig. S4. The scatter plot and fitted relationships between EcM stem proportion and tree richness without controlling for covariates in three latitudinal regions.

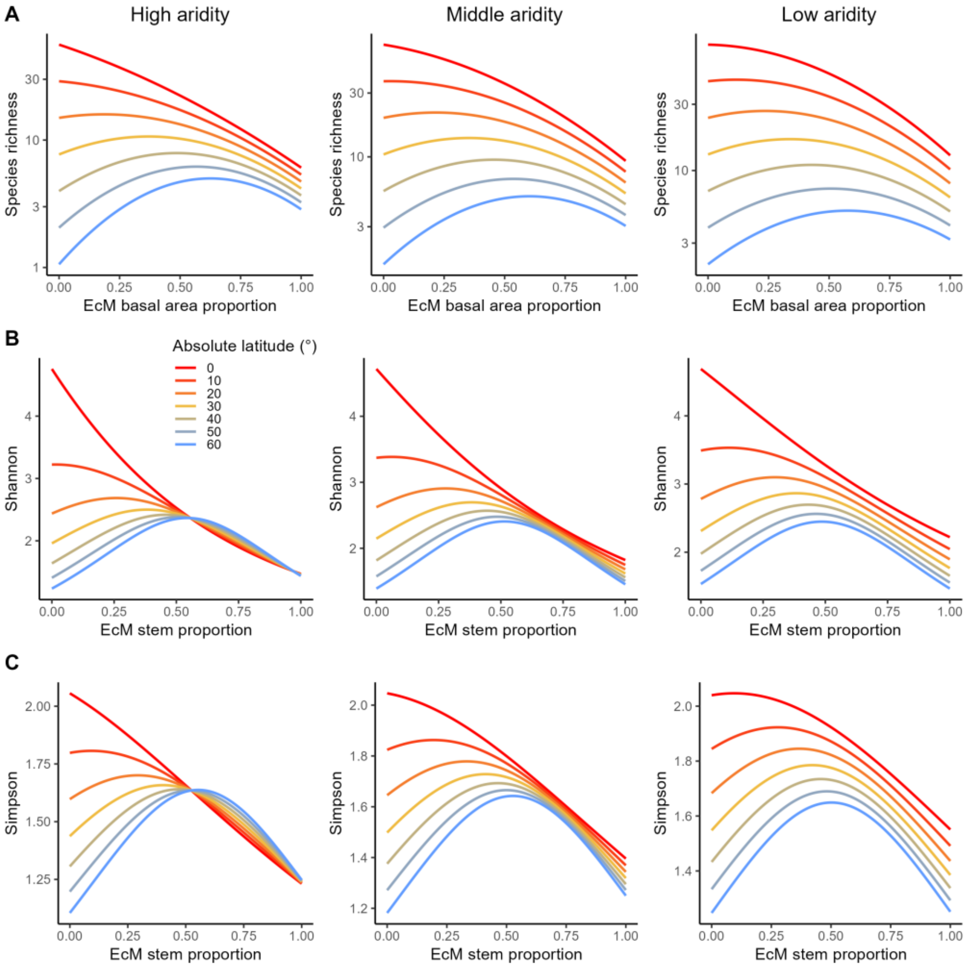

Fig. S5. The relationships between ectomycorrhizal (EcM) tree proportion and species diversity vary across gradients of absolute latitude (0-60°) and aridity index (0.5, 0.9, and 1.2) evaluated using generalized linear models. A, EcM basal area proportion instead of EcM stem proportion; **B,** Shannon index instead of species richness; **C,** Simpson index instead of species richness. Aridity index values differ slightly from those in the main text because we needed to predict reasonable ranges based on our observed values.

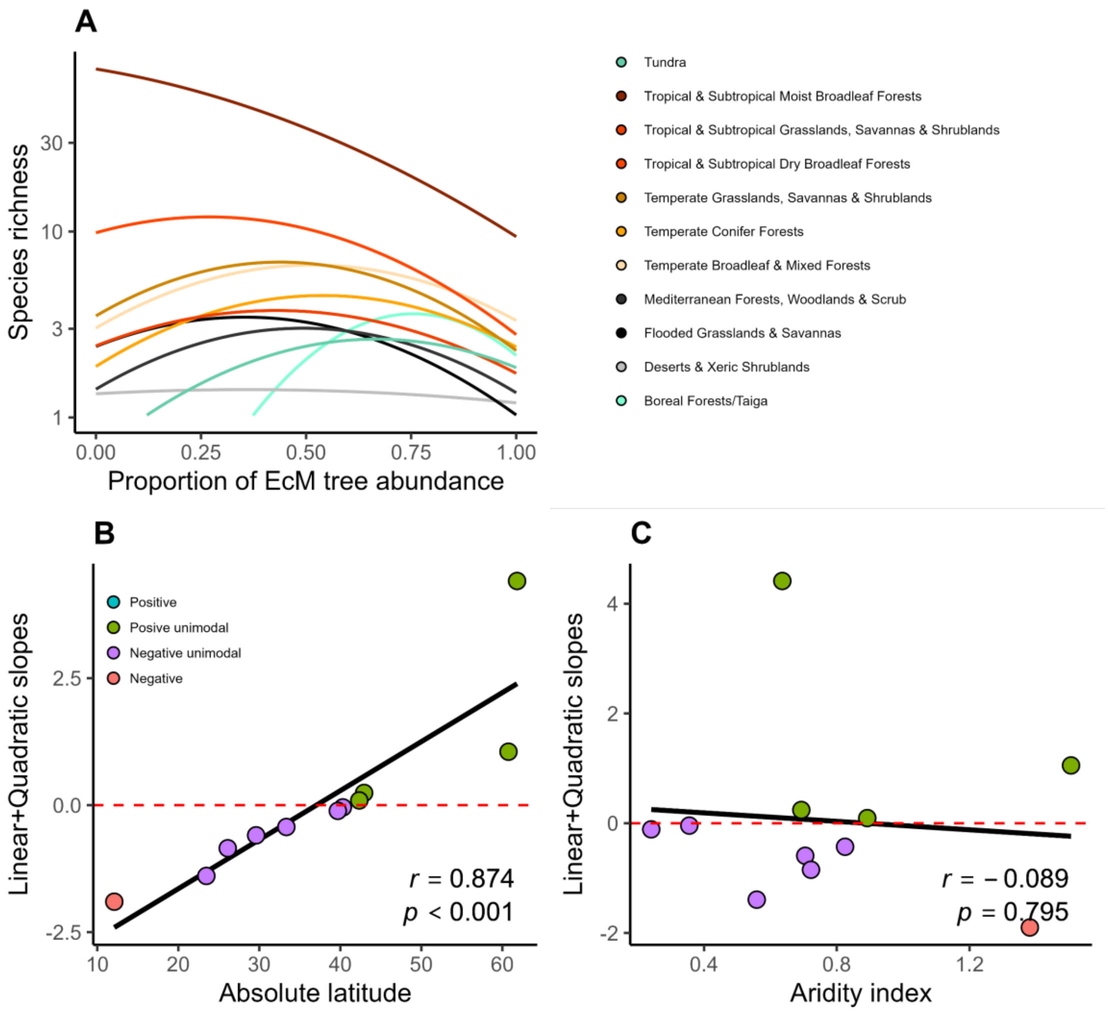

Fig. S6. The relationships between tree species richness and EcM tree proportion within biomes using the generalized linear model. These relationships consistently tend to be more negative from boreal to tropical forests evaluated. **A,** Predicted species richness along with the proportion of EcM trees. **B-C,** The relationships between the sum of slopes for linear and quadratic EcM proportion and absolute latitudes and aridity index. Especially, for the sum of slopes, negative slopes mean both linear and quadratic terms are negative; negatively unimodal slopes mean EcM+EcM2 slope is negative but EcM and EcM2 slopes have different signatures; positive unimodal slopes mean the EcM+EcM2 slope is positive but EcM2 slopes have different signatures; and positive slopes mean both linear and quadratic terms are positive. The models are performed based on all forest plot data and biomes with less than 100 sample points are excluded.

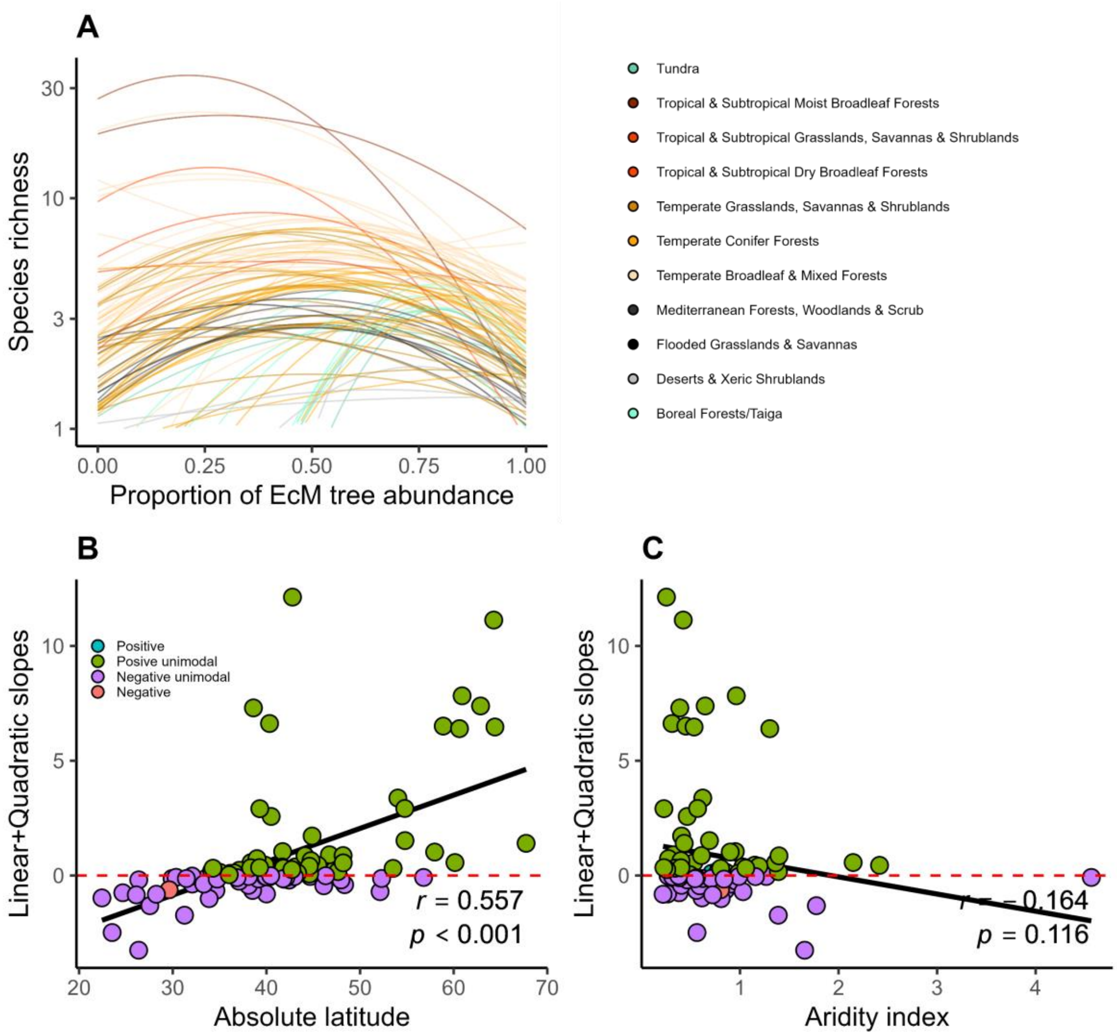

Fig. S7. The relationships between tree species richness and EcM tree proportion within ecoregions using the generalized linear model. These relationships consistently tend to be more negative from boreal to tropical forests evaluated. **A,** Predicted species richness along with the proportion of EcM trees. **B-C,** The relationships between the sum of slopes for linear and quadratic EcM proportion and absolute latitudes and aridity index. Especially, for the sum of slopes, negative slopes mean both linear and quadratic terms are negative; negatively unimodal slopes mean EcM+EcM2 slope is negative but EcM and EcM2 slopes have different signatures; positive unimodal slopes mean the EcM+EcM2 slope is positive but EcM2 slopes have different signatures; and positive slopes mean both linear and quadratic terms are positive. The models are performed based on all forest plot data and biomes with less than 100 sample points are excluded.

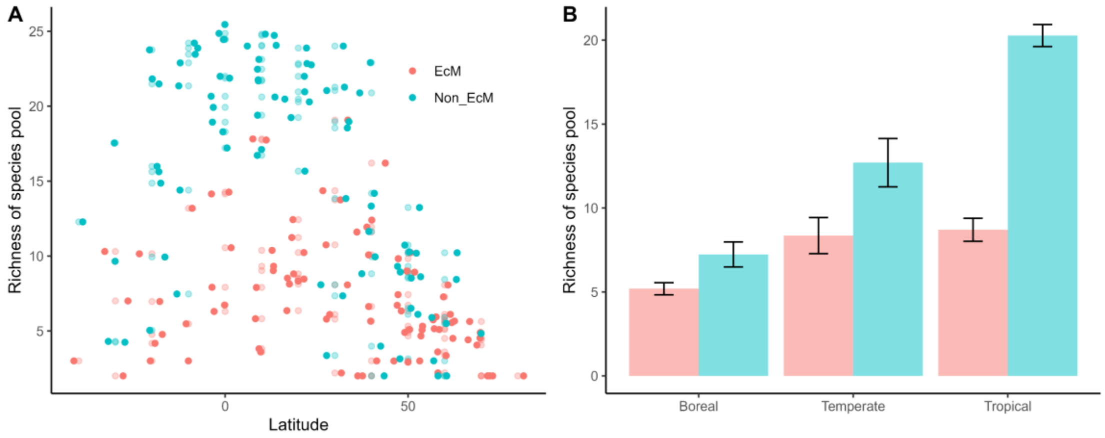

Fig. S8. Variation in the rarefied richness of EcM and non-EcM species pools across latitude. Rarefied richness was calculated using the sample of 50 tree individuals within a 10° × 10° grid across latitudes (A) and their mean values and standard error aggregated within three latitudinal regions (B). Richness has been log10-transformed.

Table S1. Model selections from six alternative models with different combinations of environments and interactions between EcM tree proportion and latitudes and aridity. The sixth model is selected as the best model with the lowest AIC value.

ID	Model	AIC	Model comparison
1	SR~(EcM+EcM2)+MAT+AI+covariates	24077	
2	SR~(EcM+EcM2)+ Lat_abs +AI+ covariates	23764	2 vs. 1: p<0.001
3	SR~(EcM+EcM2)*Lat_abs+AI+ covariates	23453	3 vs. 2: p<0.001
4	SR~(EcM+EcM2)*AI+Lat_abs+ covariates	23720	4 vs. 3: p=1
5	SR~(EcM+EcM2)*Lat_abs+(EcM+EcM2)*AI+covariates	23431	5 vs. 4: p<0.001
**6**	**SR~(EcM+EcM2)*AI*Lat_abs+covariates**	**23389**	6 vs. 5: p<0.001

Covariates: Elevation +Slope + Soil clay + Soil cec + DBH + Log (Plot area)+ Spatial autocorrelation.

SR, species richness; EcM, EcM stem proportion; AI, aridity index.

Table S2. Results for different models in our study.

Model	variable	Estimate	SE	p	lower	upper
Null	EcM	-3.906	0.166	0	-4.24	-3.57
	EcM2	0.754	0.165	0	0.413	1.093
Latitude * AI	Elevation	-0.131	0.014	0	-0.159	-0.103
	Slope	0.087	0.013	0	0.061	0.114
	Soil clay	0.12	0.011	0	0.097	0.143
	Soil cec	-0.115	0.011	0	-0.136	-0.095
	DBH	-0.347	0.013	0	-0.373	-0.321
	Log (Plot area)	0.113	0.011	0	0.091	0.134
	Spatial autocorrelation	0.39	0.007	0	0.376	0.405
	EcM	0.561	0.045	0	0.471	0.651
	EcM2	-0.952	0.05	0	-1.051	-0.852
	AI	0.375	0.021	0	0.332	0.418
	Lat_abs	-0.585	0.018	0	-0.621	-0.548
	EcM:AI	-0.039	0.052	0.4564	-0.147	0.069
	EcM2:AI	-0.047	0.058	0.4162	-0.166	0.071
	EcM:Lat_abs	0.707	0.054	0	0.598	0.815
	EcM2:Lat_abs	-0.337	0.057	0	-0.45	-0.223
	AI:Lat_abs	-0.055	0.016	5.00E-04	-0.087	-0.023
	EcM:AI:Lat_abs	-0.382	0.041	0	-0.465	-0.298
	EcM2:AI:Lat_abs	0.316	0.045	0	0.223	0.41
Basal area-based proportion	Elevation	-0.135	0.014	0	-0.164	-0.107
	Slope	0.086	0.013	0	0.06	0.112
	Soil clay	0.121	0.012	0	0.098	0.144
	Soil cec	-0.114	0.011	0	-0.135	-0.094
	DBH	-0.388	0.013	0	-0.414	-0.362
	Log (Plot area)	0.111	0.011	0	0.09	0.133
	Spatial autocorrelation	0.407	0.007	0	0.392	0.421
	EcM	0.735	0.045	0	0.645	0.825
	EcM2	-0.989	0.047	0	-1.082	-0.895
	AI	0.417	0.018	0	0.38	0.453
	Lat_abs	-0.653	0.017	0	-0.686	-0.619
	EcM:AI	-0.15	0.053	0.005	-0.26	-0.04
	EcM2:AI	0.096	0.055	0.0819	-0.018	0.209
	EcM:Lat_abs	0.497	0.051	0	0.396	0.597
	EcM2:Lat_abs	-0.2	0.053	1.00E-04	-0.305	-0.095
	AI:Lat_abs	-0.089	0.013	0	-0.117	-0.062
	EcM:AI:Lat_abs	-0.357	0.042	0	-0.445	-0.269
	EcM2:AI:Lat_abs	0.257	0.043	0	0.166	0.349
Shannon	Elevation	0.012	0.002	0	0.007	0.016
	Slope	-0.014	0.002	0	-0.018	-0.01
	Soil clay	-0.012	0.002	0	-0.016	-0.009
	Soil cec	0.016	0.002	0	0.012	0.019
	DBH	0.041	0.002	0	0.036	0.045
	Log (Plot area)	-0.006	0.002	5.00E-04	-0.009	-0.003
	Spatial autocorrelation	-0.062	0.001	0	-0.064	-0.059
	EcM	-0.222	0.008	0	-0.237	-0.206
	EcM2	0.282	0.009	0	0.265	0.3

	AI	-0.077	0.004	0	-0.085	-0.069
	Lat_abs	0.073	0.003	0	0.067	0.08
	EcM:AI	0.064	0.008	0	0.048	0.08
	EcM2:AI	-0.076	0.009	0	-0.094	-0.058
	EcM:Lat_abs	-0.153	0.009	0	-0.17	-0.135
	EcM2:Lat_abs	0.117	0.009	0	0.098	0.135
	AI:Lat_abs	0.007	0.003	0.0127	0.002	0.013
	EcM:AI:Lat_abs	0.061	0.006	0	0.049	0.073
	EcM2:AI:Lat_abs	-0.02	0.007	0.004	-0.033	-0.006
Simpson	Elevation	0.009	0.002	0	0.005	0.013
	Slope	-0.009	0.002	0	-0.013	-0.005
	Soil clay	-0.001	0.002	0.7172	-0.004	0.003
	Soil cec	0.01	0.002	0	0.007	0.013
	DBH	0.02	0.002	0	0.017	0.024
	Log (Plot area)	0.001	0.002	0.5232	-0.002	0.004
	Spatial autocorrelation	-0.058	0.001	0	-0.061	-0.056
	EcM	-0.189	0.007	0	-0.202	-0.176
	EcM2	0.225	0.007	0	0.211	0.239
	AI	-0.054	0.003	0	-0.059	-0.048
	Lat_abs	0.051	0.003	0	0.046	0.057
	EcM:AI	0.029	0.008	2.00E-04	0.014	0.044
	EcM2:AI	-0.039	0.008	0	-0.055	-0.022
	EcM:Lat_abs	-0.103	0.008	0	-0.119	-0.087
	EcM2:Lat_abs	0.076	0.008	0	0.06	0.092
	AI:Lat_abs	0.009	0.002	1.00E-04	0.004	0.013
	EcM:AI:Lat_abs	0.049	0.006	0	0.038	0.061
	EcM2:AI:Lat_abs	-0.02	0.007	0.0018	-0.033	-0.008

MAT, mean annual temperature; AI, aridity index; DBH, diameter at breast height threshold in plot survey; soil clay, soil clay content; soil cec, soil cation exchange capacity.

Table S3. Comparisons of model performance for structural equation modelling.

Data	Model type	Fisher's C	P value	AIC
Global	Soil→EcM	8.42	0.39	**32216**
	EcM→Soil	2.93	0.57	108771
Boreal	Soil→EcM	7.06	0.53	**2179**
	EcM→Soil	2.82	0.59	13161
Temperate	Soil→EcM	6.22	0.62	**17421**
	EcM→Soil	1.82	0.77	62745
Tropical	Soil→EcM	3.49	0.90	**10661**
	EcM→Soil	1.72	0.79	27999

We compared models that included either the path representing the effect of soil on EcM tree proportion (Soil → EcM) or the path representing the effect of EcM tree proportion on soil (EcM → Soil). Notably, when including the EcM → Soil path (see model structure below), the models had to be more complex to achieve a Fisher's C test p-value greater than 0.05.

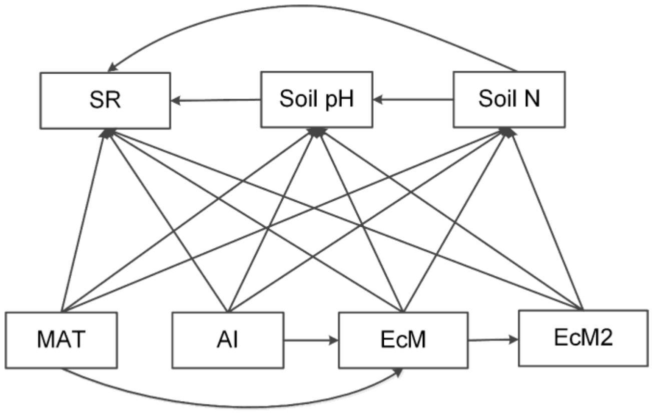

Supplement: Supplementary file 2 — Figs. S1 to S8 Tables S1 to S3 [file sciadv.adt5743_sm.pdf]
